# A de novo, mosaic and complex chromosome 21 rearrangement causes *APP* triplication and familial autosomal dominant early onset Alzheimer disease

**DOI:** 10.1038/s41598-025-86645-0

**Published:** 2025-01-23

**Authors:** Emma Ehn, Jesper Eisfeldt, Jose M. Laffita-Mesa, Håkan Thonberg, Jacqueline Schoumans, Anne M. Portaankorva, Matti Viitanen, Anna Lindstrand, Inger Nennesmo, Caroline Graff

**Affiliations:** 1 Division for Neurogeriatrics, Centre for Alzheimer Research, Department of Neurobiology, Care Sciences and Society, Karolinska Institutet, Stockholm, Sweden; 2 Unit for Hereditary Dementias, Karolinska University Hospital Solna, Stockholm, Sweden; 3 Department for Molecular Medicine and Surgery, Karolinska Institutet, Stockholm, Sweden; 4 Department of Clinical Genetics and Genomics, Karolinska University Hospital Solna, Stockholm, Sweden; 5 Département de Médicine de Laboratoire et Pathologie, Centre Universitaire Hospitalier Vaudois (CHUV), Lausanne, Switzerland; 6 Faculty of Medicine, Clinical Neurosciences, University of Helsinki, Helsinki, Finland; 7 Division for Clinical Geriatrics, Department of Neurobiology, Care Sciences and Society, Karolinska Institutet, Huddinge, Sweden; 8 Department of Oncology-Pathology, Karolinska Institutet, Stockholm, Sweden; 9 Department of Pathology and Cancer Diagnostics, Karolinska University Hospital Solna, Stockholm, Sweden

**Keywords:** Autosomal dominant Alzheimer disease (ADAD), Amyloid-β precursor protein gene (*APP*), Cerebral amyloid angiopathy (CAA), Complex genomic rearrangement (CGR), Mosaicism, Structural variant (SV)., Computational biology and bioinformatics, Genetics, Neuroscience, Clinical genetics, Chromosome abnormality, Alzheimer's disease, Genetics research

## Abstract

Copy number variation (CNV) of the amyloid-β precursor protein gene (*APP*) is a known cause of autosomal dominant Alzheimer disease (ADAD), but *de novo* genetic variants causing ADAD are rare. We report a mother and daughter with neuropathologically confirmed definite Alzheimer disease (AD) and extensive cerebral amyloid angiopathy (CAA). Copy number analysis identified an increased number of *APP* copies and genome sequencing (GS) revealed the underlying complex genomic rearrangement (CGR) including a triplication of *APP* with two unique breakpoint junctions (BPJs). The mosaic state in the mother had likely occurred *de novo*. Digital droplet PCR (ddPCR) on 42 different tissues, including 17 different brain regions, showed the derivative chromosome at varying mosaic levels (20–96%) in the mother who had symptom onset at age 58 years. In contrast, the derivative chromosome was present in all analyzed cells in the daughter whose symptom onset was at 34 years. This study reveals the architecture of a *de novo* CGR causing *APP* triplication and ADAD with a striking difference in age at onset between the fully heterozygous daughter compared to the mosaic mother. The GS analysis identified the complexity of the CGR illustrating its usefulness in identifying structural variants (SVs) in neurodegenerative disorders.

**Supplementary Information:**

The online version contains supplementary material available at 10.1038/s41598-025-86645-0.

## Introduction

Dementia is a global health burden affecting more than 50 million individuals worldwide and Alzheimer disease (AD) accounts for a majority of these cases^1^. Processing of the amyloid-β precursor protein (APP) and accumulation of amyloid-β (Aβ) peptides as plaques in the brain parenchyma is the most established AD pathogenesis model called the amyloid cascade hypothesis^2^. The neuropathologic hallmarks of AD are extracellular deposits of Aβ peptides and intraneuronal aggregates of hyperphosphorylated tau^3^. In AD-related cerebral amyloid angiopathy (CAA), Aβ-peptides are deposited in the walls of cerebral arteries and weakens the vessel walls which may result in cerebral hemorrhage^4^. The Aβ deposits in CAA, predominantly of Aβ40 type, are mainly found in the media and adventitia of cerebral arterioles in cortex and leptomeninges^5,6^. CAA with Aβ co-occur in about 50% of all cases with AD neuropathology but is also present in postmortem brains without hallmark AD-pathology^7^.

The great majority of all AD cases (90–95%) have a late onset (LOAD) after 65 years of age, while in 5–10%, the disease starts before 65 years (early onset -EOAD)^8^. Less than 1% of all AD cases (10% of EOAD) have early onset autosomal dominant Alzheimer disease (ADAD) with a disease-causing variant in amyloid-β precursor protein gene (*APP*), presenilin 1 (*PSEN1)* or presenilin 2 (*PSEN2)*. The heritability in AD is estimated to 79% in LOAD and 92–100% in EOAD, and one of the identified major risk-variants is the ε4 allele in apolipoprotein E (*APOE* ε4) but additional genetic variants remain to be discovered^9,10^. Although rare, and contributing very little to the overall heritability, detection of the deterministic genetic variants is of importance for drug development and understanding the AD pathogenesis and allows for counselling of patients and families about risk, predictive (carrier) testing and preimplantation genetic testing (PGT)^8^.

Pathogenic/likely pathogenic single nucleotide variants (SNVs) and insertion/deletions (indels) are the most common genetic changes in *APP*, *PSEN1* and *PSEN2* causing ADAD. In addition, both *APP* duplications and triplication have been shown to cause ADAD^11–15^. Furthermore, trisomy 21, the main underlying genetic background in Down syndrome, is associated with an almost 100% life time risk for genetic AD due to a copy number increase of *APP* which is located on chromosome 21^16^. Interestingly, CAA is a common finding in *APP* duplications and in trisomy 21, but in the latter case, CAA more rarely results in intracerebral hemorrhage (ICH)^17,18^. In addition, several SNVs in *APP*, including the Dutch, the Italian, the Iowa, the Greek, the Flemish, the Swedish and the Arctic variants among others, are associated with CAA with or without AD pathology^19^.

Structural variants (SVs) are a group of genetic changes that affect the structure of the DNA molecule including losses (deletions), gains (duplications and triplications), inversions and complex genomic rearrangements (CGRs) encompassing two or more breakpoint junctions (BPJs)^20^. Recent work has demonstrated the contribution of SVs to genetic disease, and each single human genome contains up to 20 000 SVs, depending on annotation and tools for SV detection^21^. Some CGRs are recurrent, probably caused by the same mechanism of origin, and in non-recurrent cases a BPJ analysis can give clues into the specific underlying mechanisms involved^21^.

Another genetic phenomenon which influences human disease is mosaicism. Mosaicism is the result of a postzygotic *de novo* event resulting in two genetically different cell lines originating from the same fertilized egg. Timing, cell type of origin and the distribution and level of mosaicism determine the outcome on phenotype^22^. A classification system of mosaicism, based on six different attributes (A-F), was recently proposed and includes: **A**ffected tissue, **B**ody pattern, **C**hange of direction, **D**evelopmental mechanism, **E**tiology and **F**raction of affected tissue^23^. Any genetic change, including aneuploidies, SVs and SNVs, can theoretically be mosaic while some genetic conditions (trisomy 8, 9, 14, 17 and 22 and McCune Albright syndrome with variants in *GNAS* gene*)* are only compatible with survival in mosaic state^24,25^. Furthermore, different levels of mosaicism can be associated with variable severity of symptoms and ages at onset^24^.

This study includes a mother and her daughter with onset of cognitive and neurological symptoms at ages 58 and 34 years respectively, which was later neuropathologically confirmed as definitive AD with CAA. Genetic analysis revealed an increased number of copies of *APP* in both subjects and there were indications that they had different doses of *APP*. Genome sequencing (GS), digital droplet PCR (ddPCR) and advanced bioinformatic analysis was applied to resolve the CGR structure, characterize its distribution and showed that it was mosaic in the mother. Additionally, we outline the mechanism of origin underlying the chromosome 21 CGR that leads to an increased *APP* copy number causing familial EOAD.

## Results

### Clinical background

Subject III:3 (the daughter), a previously healthy individual, presented with left-sided weakness at 34 years of age and signs of right-sided, subcortical intracerebral hemorrhage was detected on magnetic resonance imaging (MRI) and computer tomography (CT) scans. Except for memory deficits, with initial mini mental test (MMT) score of 23–25, there were no other sequel. A follow-up MRI was done two years later, due to progressive memory impairment, showed a large number of micro bleedings in both cerebral hemispheres. At this time the imaging suggested CAA and although she was not formally diagnosed with AD, she received Rivastigmine therapy. Seizures, myoclonus and spasticity presented at age 37 in combination with progressive cognitive deterioration, global aphasia and fluctuating consciousness. At this time MRI showed more extensive micro bleedings also involving the occipital cortex and white matter changes. Electroencephalogram (EEG) did not show any epileptic activity, and she did not present any focal neurologic signs but due to repeated seizures, she received carbamazepine and steroid therapy. CSF markers were negative for syphilis and borrelia, but the CSF/serum albumin ratio was slightly increased, indicating a blood brain barrier dysfunction although no cells were detected. CSF AD biomarkers were not analyzed. At age 38, another large right-sided intracerebral bleeding caused hemiplegia and unconsciousness, which required surgical intervention and resulted in persistent, left-sided paresis, and repeated seizures. Ten years after the initial symptoms, at the age of 44, she was bedridden and died of pneumonia in a nursing home and a neuropathological examination was performed postmortem.

Subject II:2 (the mother) retired early due to chronic pain in neck and shoulders caused by a previous whiplash injury at 51 years of age. Her next of kin noticed memory and attention deficits in combination with word finding problems starting at 58 years of age, at the time when her daughter was already severely cognitively impaired. She also complained about repeated headaches. Evaluation at the memory clinic at age 62 revealed memory and language problems with Mini Mental State Examination (MMSE)-score 24/30 but standard neurologic examination was normal. An MRI showed a suspected older bleeding (0,5 cm) in the left frontal hemisphere and signs of older bleedings in the frontal left parenchyma, deep down in a sulcus. No white matter changes were seen but a 1 cm meningioma was observed on the surface of the right frontal lobe. Neuropsychological examination revealed markedly affected verbal abilities including word finding problems, light visual perception difficulties, impaired short term and working memory but intact visuospatial abilities, indicating an overall cognitive impairment. Speech therapist assessment showed affected speech fluency, word generation and speech motor control. At follow-up one year later, her MMSE was 20/30 and she had daily headaches. A second brain MRI showed no changes compared to the previous examination whereas Single-Photon Emission Computerized Tomography (SPECT) with Technetium-99 m hexamethyl propylene amine oxime (Tc-99 m-HMPAO) showed decreased uptake in the temporo-parieto-occipital regions on the left side. CSF biomarkers, analyzed within the clinical diagnostic routine, were pathological with increased total-tau 710ng/uL (ref < 400ng/L) and P-tau 93ng/L (ref < 60ng/L). Unfortunately, β-amyloid-42 could not be provided because CSF was collected in the wrong sampling tube. She was thereby diagnosed with atypical/mixed AD and received Galantamine therapy. She passed away nine years later at age 72 years and a neuropathological examination was performed postmortem.

### Family history

**Fig. 1:**
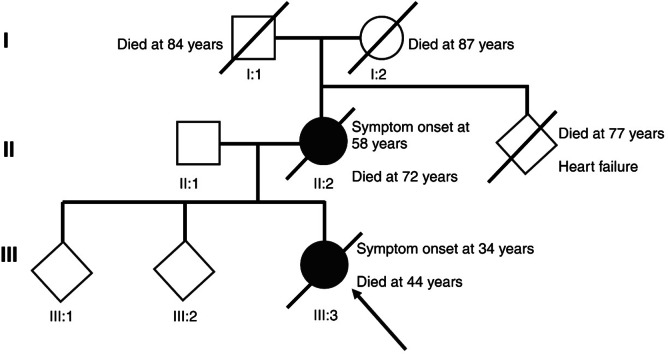
Pedigree Subjects II:2 and III:3, with symptom onset at age 58 and 34 years respectively. There were no reports of cognitive impairment in the parents of subject II:2 and they both lived beyond age 80 years. The proband, subject III:3 depicted by an arrow, had two siblings without cognitive impairments. Pedigree drawn in BioRender at https://www.biorender.com.

There were no reports on any other family members with dementia or cognitive decline. The father and mother of subject II:2 did not show any symptoms of dementia and died at age 84 and 87 years respectively. Subject III:3 had two healthy siblings, and all three were born during the same decade (Fig. 1).

### Neuropathological examinations

#### Subject III:3

**Fig. 2:**
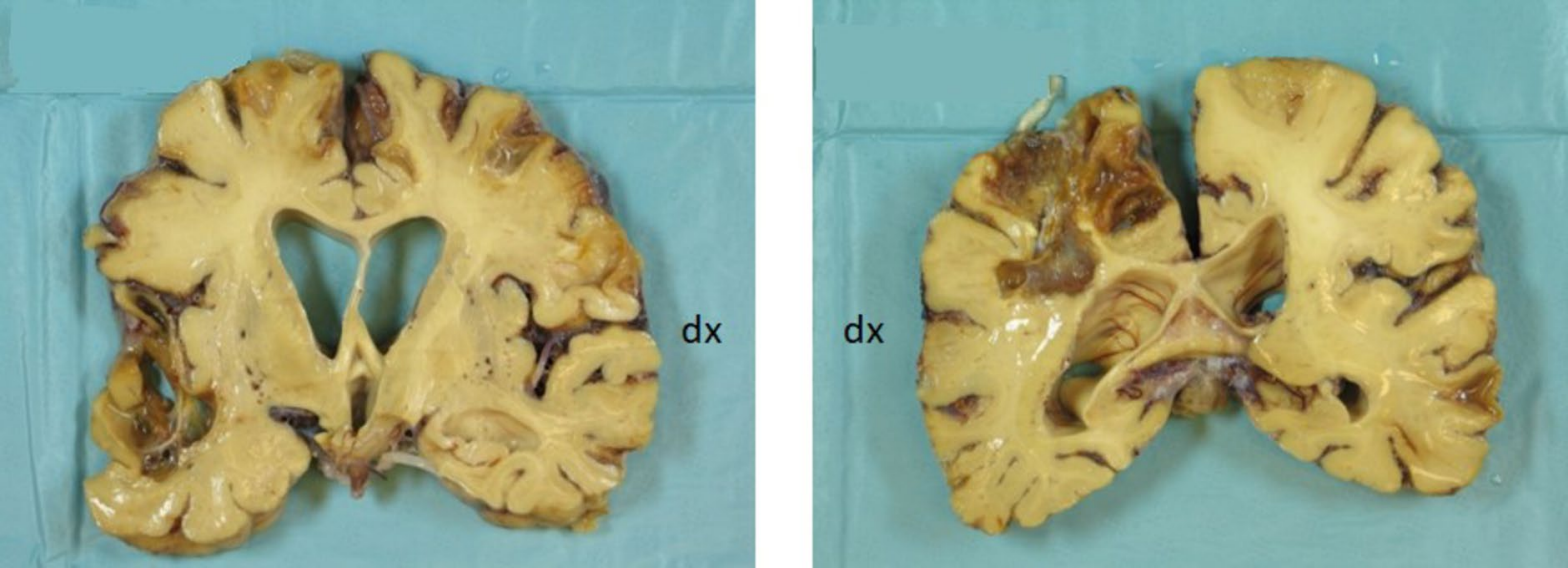
Macroscopic brain images in subject III:3. Extensive brain lesions due to hemorrhages.

The brain weighed 840 gram, the cerebellum 120 gram of that. The gyri were small, especially in the frontal and parietal lobes. The meninges were thickened. On coronal sections prominent loss of parenchyma was seen in the superior right frontoparietal lobes near the midline and in the left anterior temporal lobe (Fig. 2). The surrounding tissue was in a yellow-brown color. Several similar but smaller lesions were found mainly superficially in the brain parenchyma. The cortex had a reduced thickness, and the lateral ventricles were dilated. The hippocampus, basal ganglia and thalamus had a slightly reduced size. The brainstem and cerebellum were macroscopically normal. The white matter of the brain had a somewhat rubbery texture.

**Fig. 3:**
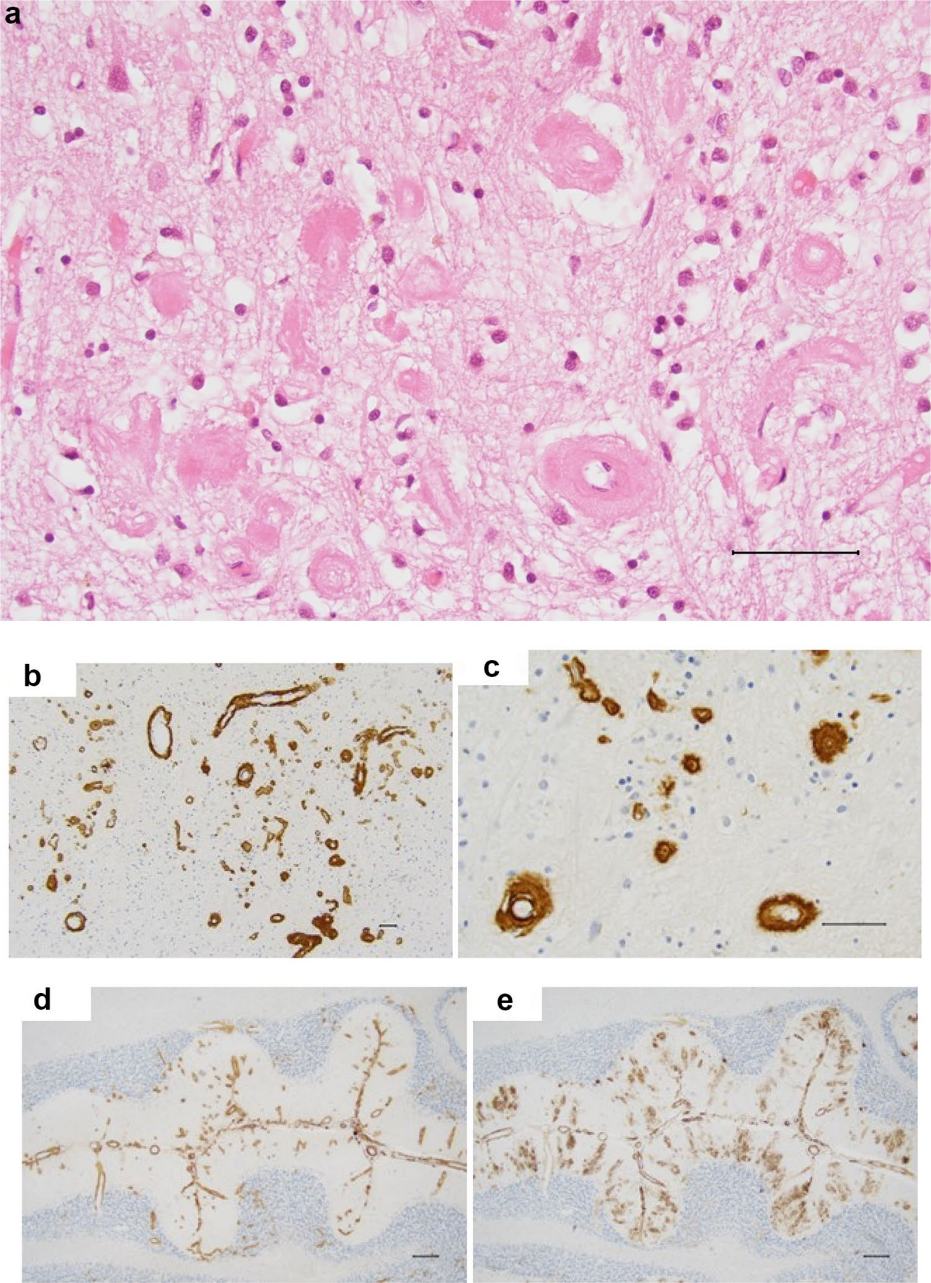
(**a**) Microscopic haematoxylin and eosin slide in subject III:3. Thickened vessel walls in the cerebral cortex (scale bar 50 μm). (**b**,**c**) Microscopic beta-amyloid 1–40 slides in subject III:3. Cerebral cortex with numerous vessels positive for staining with beta-amyloid antibodies 1–40 (scale bars 50 μm). (**d**,**e**) Sections of the cerebellum from subject III:3 Stained with antibodies against beta-amyloid 1–40 (**d**) and 1–42 (**e**) (scale bars 200 μm).

Microscopically, several additional small destructions were found in the parenchyma with the presence of hemosiderin laden macrophages. In the cerebral cortex there was widespread severe loss of neurons. In better preserved regions numerous neuritic plaques, many with a central core, as well as neurofibrillary tangles were present in silver-stained sections. The walls of the blood vessels were thickened and amorphous (Fig. 3a). In polarized light Congo-stained sections showed apple-green birefringence. For antibodies against beta amyloid 1–40 positivity in some plaques but mainly in vessels of different size, from large mostly in the leptomeninges to medium sized and capillaries in the cortex, was found (Fig. 3b) with microscopic picture of dyshoric capillary angiopathy (Fig. 3c). In larger vessels threadlike extensions of beta-amyloid from the outer wall into the neuropil were detected. In the larger vessels in the leptomeninges immunoreactivity was mainly present in the adventitia while in the cortex positivity was observed throughout the wall of the vessels. For beta 1–42 antibodies immunopositivity was also found in vessels but to a lesser degree than for beta 1–40. For beta 1–42 many plaque structures, both diffuse and neuritic, some with a central core, were noticed. In the hippocampus some loss of neurons was seen. The silver-stained section showed many neurofibrillary tangles and neuritic plaques. In the amygdala many small cavities were present. Some loss of neurons was found in the putamen and thalamus but there was no clear presence of plaque structures. There was a loss of pigmented neurons in the substantia nigra in section from the mesencephalon. Degeneration of the right cortico-spinal fibers was seen in the mesencephalon and in pons and the right pyramid in the medulla oblongata was small. In the cerebellum, beta amyloid 1–40 showed numerous positive vessels in the leptomeninges and cortex while beta amyloid 1–42 staining also revealed amyloid deposits in the molecular layer (Fig. 3d and e). Prominent tau pathology was seen in the cerebral cortex with positivity in the cytoplasm of neurons and in the neuropil. In sections stained with Luxol fast blue there was severe loss of myelinated fibers in the white matter of the brain. The ABC score, describing AD neuropathology advancement including abeta plaque score (A), neurofibrillary tangle score (B) and neuritic plaque score (C) was A3B3C3. The CAA was of both type 1 and type 2 i.e. amyloid deposits in capillaries (type 1) or larger vessels (type 2)^3^.

#### Subject II:2

The left half of the brain was frozen at autopsy and the right fixed in formaldehyde for histological examination. The right cerebral hemisphere weighed 400 gram and the right cerebellar hemisphere 60 gram. The frontal gyri had reduced size. On sections of the brain, the cortex showed some reduction in thickness. The hippocampus was slightly smaller than usual. The basal ganglia and thalamus as well as the brainstem and cerebellum were macroscopically normal. No hemorrhages were present in the brain parenchyma.

Microscopically, in the cerebral cortex loss of neurons was seen mainly in the superficial layers with vacuolization of the tissue, most prominent in the frontal lobe. In silver staining numerous diffuse and neuritic plaques, many with a central core, were found in the cortex of the brain. Mainly diffuse but also some neuritic plaques with a central core were also present in the basal ganglia and thalamus. In the molecular layer of the cerebellum diffuse plaques were found. Neurofibrillary tangles were detected in most cortical regions, but they were much fewer compared to the number of plaques. In the hippocampus neurofibrillary tangles and some plaques were seen. In Congo red-stained sections many positive vessel walls, mainly in the leptomeninges, were found. For some of them only a part of the circumference stained positively. Some vessels, in especially the cortex of the occipital lobe, were surrounded by a lot of amyloid. The presence of CAA was, however, far less prominent in II: II than in III: III and obvious capillary positivity was not seen. The substantia nigra in the mesencephalon was well preserved. The ABC score was A3B2C3. The CAA was of type 2.

### Array comparative genome hybridization

Array-CGH on DNA from blood showed an increased copy number of a region on chromosome 21 including the *APP* gene, and subject III:3 appeared to have more copies compared to subject II:2 as illustrated by a difference in mean log2 ratio (mean log2 ratio 1.0 = 4 copies, mean log2 ratio 0.585 = 3 copies). The inner boundaries of the copy number increased region were chr21:26,887,899 − 27,948,734. Hence, the size of the copy number increased region (1.06 Mb) was different from and larger compared to the Finnish (0.713 Mb) and Swedish (0.947 Mb) duplication cases described previously^15,26^ (see Supplementary Fig. S1).

### Genome sequencing

**Fig. 4:**
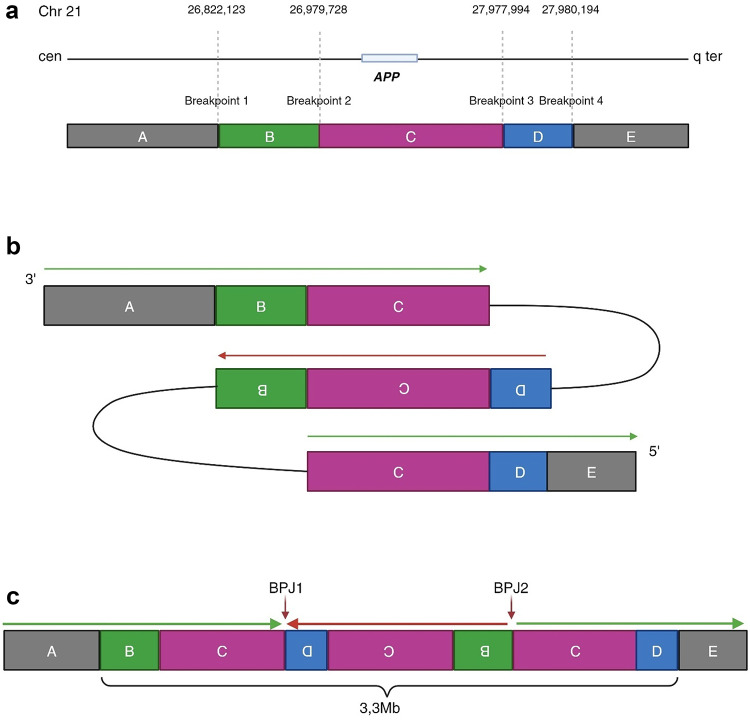
Reference genomic positions. Breakpoints 1–4 in segments A, B, C and D. The location of the *APP* gene is annotated with a blue horizontal box and is within segment C. (**b**) Schematic illustration of the complex chromosome 21 rearrangement. The middle section, containing segment D, C and B, is in an inverted orientation (red arrow), creating two unique breakpoint junction (BPJ) sequences (black curved lines). (**c**) Schematic illustration of the derivative chromosome. Vertical red arrows show the two breakpoint junctions (BPJ1 and BPJ2). Green arrow depicts forward read direction and red arrow reverse read direction. Segment C is triplicated and so is the *APP* gene within this segment, resulting in an *APP* triplication. Illustrations created with BioRender at https://www.biorender.com.

Detailed bioinformatic analyses of GS data from both subjects revealed a CGR on chromosome 21, spanning in total 3,3 Mb (Fig. 4). The rearrangement contains four breakpoints and includes a triplicated region (segment C, position chr21:26,979,728-27-977,994) flanked by two duplicated regions (segment B, position chr21:26,822,123 − 26,979,728 and segment D, position chr21:27,977,994 − 27,980,194). For IGV details of the GS analysis, see Supplementary Figure S2. The triplicated region (segment C) contains five genes: *JAM2* (OMIM#606870), *ATP5PF/ATP5J* (OMIM#603152), *GABPA* (OMIM#600609), *APP* (OMIM#104760) and *CYYR1* (OMIM#616020) while one of the duplicated regions (segment B) contains two genes: *MIR155* (OMIM#609337) and *AP000223.42* (*LINC00515*). The middle segment of the rearrangement (one copy of segment D, C and B respectively) is in inverted orientation, generating two unique breakpoint junctions (BPJ1 and BPJ2). One gene is disrupted by the inversion in BPJ2: *MRPL39* (OMIM#611845). Haplotype analysis revealed that all involved segments are in *cis* configuration and the read depth indicates a mosaic state in subject II:2. Both subjects were *APOE* Ɛ3 homozygous and no disease-causing SNV or short tandem repeat (STR) was found in the neurodegenerative gene panel, including *APP*, *PSEN1* and *PSEN2*. See genes included in the neurodegeneration gene panel v8 in Supplementary Figure S3. Re-sequencing with Sanger methodology in subject III:3 verified the BPJ sequences from the GS. See Supplementary Figure S4 for BPJ1 and BPJ2 sequencing results.

### Breakpoint analysis

**Fig. 5:**
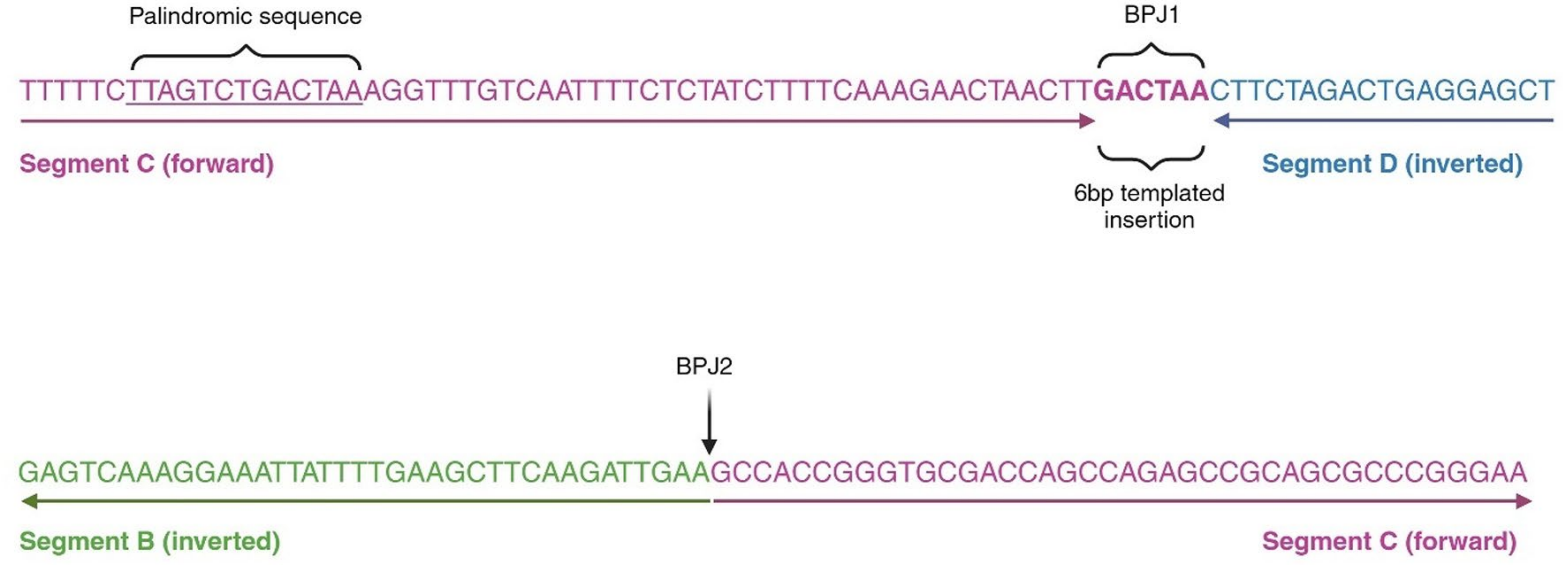
Breakpoint junction sequences. Sequences flanking and in breakpoint junctions BPJ1 and BPJ2. Arrows are pointing in the read direction. Segment C is shown in pink letters and segment D in blue. Bold pink letters in BPJ1 depict a 6 bp templated insertion originating from the palindromic sequence (underscored pink letters) 40 bp upstream of BPJ1. Of note, both sides of BPJ1 are flanked by identical CTT sequences. The blunt BPJ2 (black arrow) is located at the border between segment B (green letters) and segment C (pink letters). Illustration created with BioRender at https://www.biorender.com.

The genomic rearrangement on chromosome 21 results in two BPJs (Fig. 5). BPJ1 links the triplicated segment C (forward) and the duplicated segment D (inverted). 40 nucleotides upstream of BPJ1, the reference sequence contains a palindromic sequence and BPJ1 contains a templated 6 bp insertion originating from the palindromic sequence. The blunt BPJ2 links segment B (inverted) and segment C (forward). Further analysis of the four breakpoint positions using the UCSC genome browser revealed that breakpoint 1 is located within a Long Terminal Repeat (LTR, LTR16A, family: ERVL) and breakpoint 2 is located within the first exon of *MRPL39*. Breakpoint 3 is within a Long Interspersed Nuclear Element (LINE) and breakpoint 4 is also in an LTR (see Supplementary Fig. S5).

### ddPCR

**Fig. 6:**
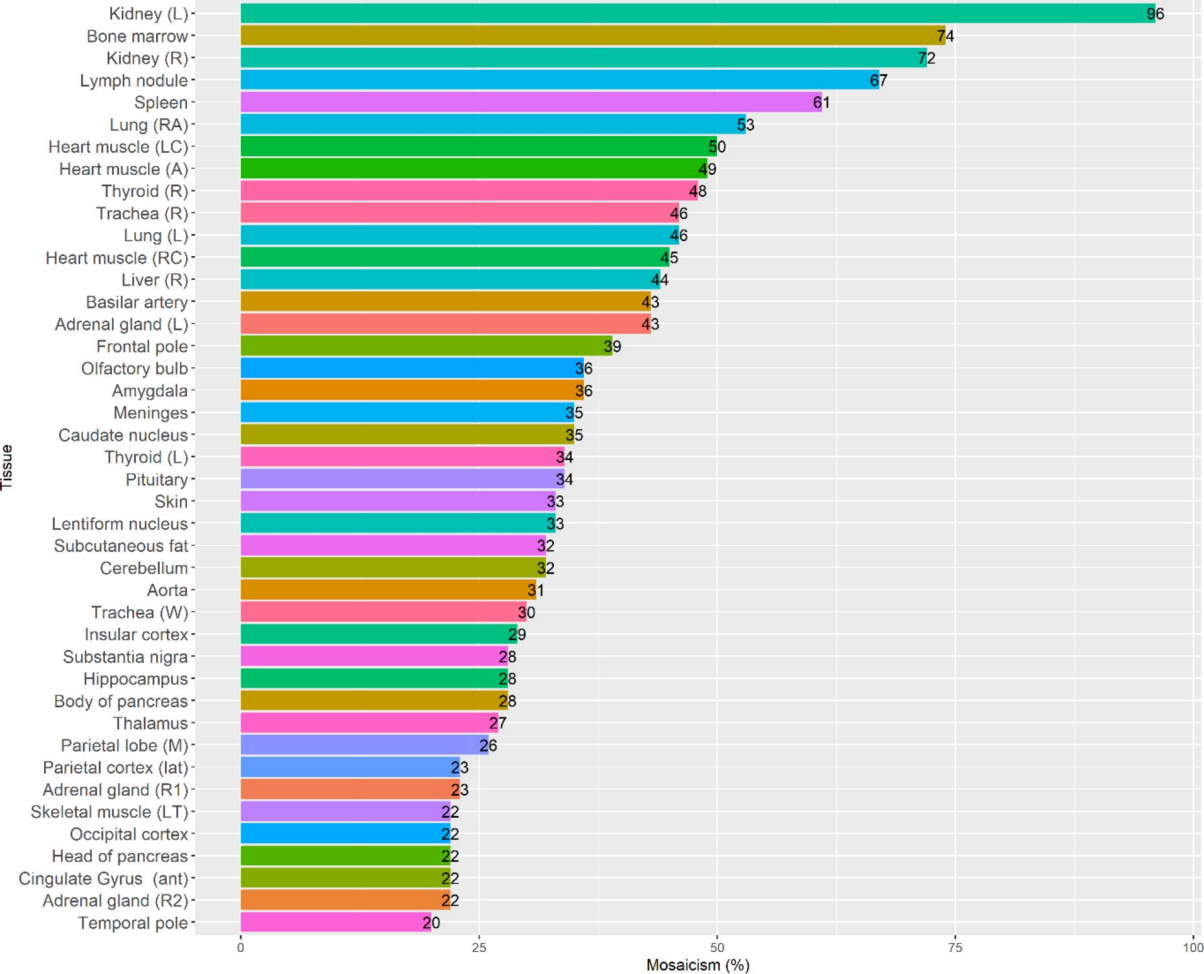
Mosaicism in all 42 analyzed tissues. Mosaic levels range from 20–96%. L = left, R = right, RA = right apical, LC = left chamber, A = atrium, RC = right chamber, W = white part, M = medial, lat = lateral, LT = left thigh, ant = anterior. Bar plot created in RStudio Version 1.3.1093.

**Fig. 7:**
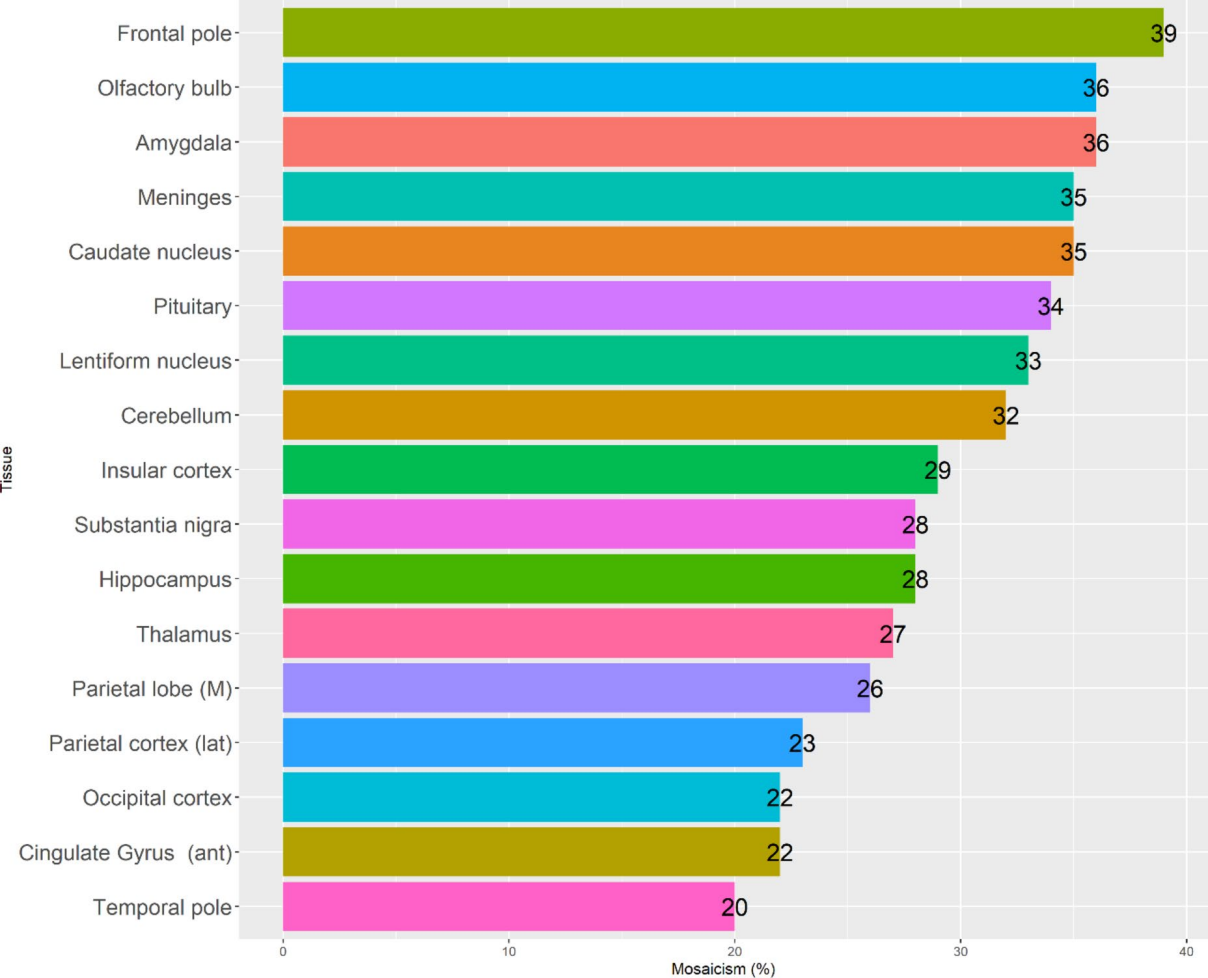
Mosaicism in all 17 analyzed brain tissues from the left side. Mosaic levels range from 20–39%. ant = anterior, lat = lateral, M = medial. Bar plot created in RStudio Version 1.3.1093.

ddPCR on available tissues from subject II:2 and III: 3 was used to quantify the level of mosaicism of the CGR (see Supplementary Table S1 for ddPCR raw data, VAF and mosaic values). Positive droplet signals for BPJ2 were detected in all 43 tissues from subject II:2, except for fascia left thigh which was excluded from further analysis (Fig. 6). The tissue with the lowest level of mosaicism was the left temporal pole of the brain (20%) and the tissue with the highest mosaicism was left kidney (96%). Mosaicism in blood was 50% (not shown). The highest brain mosaic value was found in the left frontal pole (39%) while the rest of the brain areas had mosaicism between 20 and 36% (Fig. 7). For some tissues, samples from both left and right side of the body were available (kidney, thyroid, heart muscle chamber, lung). See Supplementary Figure S6 for bar plot of left and right-side mosaicism. Mosaic level from some tissues differed rather much between body sites (thyroid right – 48%, thyroid left 34%, kidney right – 72%, kidney left 96%, adrenal gland right (R2) – 22%, adrenal gland left 43%), whereas body site mosaicism was quite similar in other tissues (lung right apical – 53%, lung left 46%, heart muscle right chamber – 45%, heart muscle left chamber – 50%). For other tissues, pieces from the same organ, but at different sites, were available for mosaicism comparison (trachea, pancreas, heart muscle, adrenal gland). See Supplementary Figure S7 for bar plot comparing mosaicism within the same tissue. In trachea, DNA was isolated from connective tissue (“white”) and smooth muscle (“red”), probably reflecting different cell types. Mosaicism for these different sites in the trachea sample was 30% (white) and 46% (red). Heart muscle right and left chamber and atrium had about the same mosaic levels (45%, 50% and 49% respectively) while adrenal gland right 1 (R1) and adrenal gland right 2 (R2) had similar but lower mosaic levels (23% and 22% respectively) compared to adrenal gland left (43%). Among the body organs, only skeletal muscle (left thigh), body of pancreas, trachea white, aorta, subcutaneous fat, skin and left thyroid had mosaicism below 40%. See Supplementary Fig. S8 for comparison of ddPCR results between tissues with high and low mosaic levels in 1D and 2D plots. In subject III:3, the CGR was found in 100% of cells in the cerebellum and blood, which were the only available tissues for analysis, reflecting a non-mosaic state.

In conclusion, the unique BPJ2 sequence was detected in all 42 tissues from subject II:2 with varying mosaic levels (20–96%). In general, tissue from brain regions had lower mosaic levels (20–39%) compared to other body organs, with the frontal pole being the brain region with the highest mosaic level (39%). Left kidney had the highest mosaicism of all tissue (96%). Cerebellum DNA from subject III:3 had the structural rearrangement in all cells (100%) compared to 32% in cerebellum from subject II:2.

## Discussion

In this study we describe a *de novo APP* triplication in a mosaic individual who transmitted the triplication to the daughter resulting in a fully heterozygous state. Both the mother and the daughter developed neuropathologically confirmed AD but their disease courses were dramatically different with a more severe phenotype in the daughter including widespread CAA and multiple ICHs.

The mosaicism in subject II:2 was classified according to the six attributes A-F^23^. The Affected tissue was of gonadosomatic type (A3; transmitted to subject III:3). The Body pattern was nonsegmental, disseminated (B2; mosaic cells in all tissues investigated). The Change of direction was benign to pathogenic mosaicism (C1) and the Developmental mechanism belonged to the type 1 segmental mosaicism (D1; postzygotic, heterozygous change in a single cell, with a risk for gonadal involvement). The Etiology of the mosaicism was a large genomic variant (E1) and the Fraction of affected tissue severe to very severe (F3-F4) (mosaic levels 20–96%). The presence of mosaic cells in all the tissues investigated suggests an early embryonic event involving all three germ layers (ectoderm, mesoderm, endoderm). Mosaic cells were found in all 17 brain regions analyzed (20–39%) and we hypothesize that the most critical factor for the development and age at onset of EOAD is the level of *APP* triplication mosaicism in brain. Although rare, genetic variants in mosaic state causing EOAD have been reported in the literature. A 52-year old woman with parkinsonian symptoms, dementia and spastic paraparesis carried a *de novo*, mosaic (8% in lymphocytes and 14% in cerebral cortex) pathogenic variant in *PSEN1*, while her daughter, with symptom onset at age 27, was fully heterozygous^27^. Furthermore, single cell analyses have detected mosaic *APP* CNV in sporadic AD, but these findings may be hampered by exogenous contamination, and thus the results on the contribution of *APP* CNV in sporadic AD is still unclear^28–30^. In yet another study, severity of the phenotype in trisomy 21 correlated to the level of mosaicism in the investigated tissue (lymphocytes and buccal swab)^31^.

In non-mosaic *APP* duplications (and hence three *APP* copies per cell), the age at onset for dementia and ICH was 42–59 and 53–64 years respectively^17^. There is one previously reported *APP* triplication (corresponding to *fourAPP* copies per cell), with symptom onset (memory deficit or transient loss of consciousness) at age 37. His father had biopsy proven CAA and probable AD with onset at 39 years. In addition, they also had ICH, seizures, and severe CAA^15^. Likewise, both subjects in our study had CAA upon neuropathological examination, but it was more extensive in subject III:3.

The delayed symptom onset at age 58 years in the mother compared to 34 years in the daughter, is therefore in line with the hypothesis that increasing levels of mosaicism correlate with an earlier age at symptom onset. The earlier age at onset (34 years) in the fully heterozygous (corresponding to 100% mosaicism) daughter is similar to the age at onset in the previously reported *APP* triplication family^15^. All reported *APP* duplications have described a similar phenotype irrespective of the size of the duplicated segment, and therefore it can be speculated that *APP* is the major copy number-sensitive gene in the region^12,17,32^. Furthermore, the neuropathological ABC score and CAA burden were more advanced in the daughter (A3B3C3, CAA type 1 and 2 ) compared to the mother (A3B2C3, CAA type 2). Additional phenotypic differences in the subjects include the absence of ICH in the mother, while the daughter suffered from extensive bleedings. We therefore speculate that these distinctions in age at onset, phenotypic presentation and severity of neuropathologic findings could be explained by the lower *APP* burden due to the mosaic state in the mother. Interestingly, the prevalence of ICH is lower in individuals with trisomy 21 compared to *APP* duplications, and it has been suggested that other genes on chromosome 21 are protective against ICH^18,33^. Parallels can be drawn with the reported duplication and triplication of the *SNCA* gene in a large family with Parkinson disease (PD), where a younger age at onset and more severe symptoms were described in the branch of the family with *SNCA* triplication^34^.

The mosaic state of the *APP* triplication in subject II:2 depicts a *de novo* genetic event in this family. *De novo* causes in ADAD seem to be rare, but are probably underdiagnosed, since genetic screening is seldom performed when family history is negative^35^. Interestingly, one study confirmed *de novo* variants (SNVs in *PSEN1* and an *APP* duplication) in 10/18 cases of apparent sporadic, early onset (under 51 years of age) AD cases^26,36^. It is of importance to identify these *de novo* cases and we suggest that individuals with very early onset dementia and/or CAA (< 40–50 years of age), even without any family history, are genetically screened with GS.

It can be speculated that the copy number increase of *APP* and other genes within the genomic rearrangement affect the functions of other organs than the brain. The APP protein is expressed outside the brain, including thymus, heart, muscle, lung, kidney, adipose tissue, liver, spleen, skin and intestine, and mRNA expression of *APP* is even more widespread (https://www.proteinatlas.org)^37^. mRNA levels of *APP* in whole blood was previously shown to be increased in *APP* triplication compared to controls similar to blood from carriers of *APP* duplications^15^. In future studies, it would be interesting to correlate the level of mosaicism and levels of *APP* mRNA from the available tissues from subject II:2 and III:3.

In our study, *JAM2*, *ATP5PF*, *GABPA* and *CYYR1* were also triplicated, but only *JAM2* has an OMIM phenotype (OMIM#618824). Biallelic loss of function (LOF) variants in *JAM2* are associated with basal ganglia calcification, but to our knowledge, there are no reports on gain of function mechanisms for *JAM2*, which is in line with of the absence of signs of brain calcification at neuropathological examination in our study^38,39^. Furthermore, the *MRPL39* gene is disrupted in BPJ2 (between segment B and C), and biallelic pathogenic variants in *MRPL39* were recently associated to a human disease phenotype (pediatric onset mitochondrial disease) for the first time^40^. However, heterozygous *MRPL39* disruption, as in our case, has not been associated with any human disease. Moreover, in the Panther database (19.0), none of these genes were found to share the same pathway as *APP* (https://www.pantherdb.org/),

The structure of the complex rearrangement (DUP-TRIP/INV-DUP) has been observed in other genes associated to human disease^21,41,42^. In one study, DUP-TRIP/INV-DUP rearrangements resulted in triplication of *MECP2* and *PLP1* through a replication-based mechanism due to the presence of inverted LCRs^42^. However, none of the breakpoints in our study are in an LCR region. Instead, breakpoint 3 is within a LINE sequence that can give rise to recombination errors and rearrangement formation^21^. On the other hand, BPJ1 contains a novel 6 bp templated insertion likely originating from an upstream palindromic structure, increasing the risk for hairpin loop formation during replication. Interestingly, both templated insertions and such harpin structures are signatures of the replication based mechanisms Fork Stalling and Template Switching (FoSTeS)/ microhomology-mediated break-induced replication (MMBIR), and we hypothesize that a combination of FoSTeS and MMBIR gave rise to the rearrangement in our study^43^. It is also noteworthy that breakpoint junction analysis in another *APP* duplication also proposed FoSTeS as the underlying mechanism^44^.

The advent of GS of patients in a clinical setting allows for efficient and large scale screening and identification of SNVs, SVs and repeat expansions^45^. We speculate that SVs underlying neurodegenerative diseases are underdiagnosed, and that GS will allow more patients to get a genetic diagnosis. Importantly, BPJ1 was in a non-coding region and Exome Sequencing (ES) or targeted gene panel analysis would probably have detected the copy number increase, but to resolve the complex architecture of the rearrangement, GS would be required. A neuropathological examination revealing extensive beta-amyloid positive CAA with ICH, can also be an indicator of on an underlying CNV in *APP*, and we emphasize that individuals with a clinical diagnosis of (early onset) dementia are offered neuropathological examination postmortem. Available postmortem brain tissue may also be used for detection of brain-specific mosaicism.

The limitations of this study include analysis of bulk DNA from large pieces of tissue, including a mix of cells from different germ layers, not reflecting the mosaic state in one pure cell type. In addition, ddPCR was only performed on tissue from the left side of the brain, since the neurodegeneration protocol that was used only covered unilateral sampling of frozen tissue. Therefore we do not know if there were any differences in levels of mosaicism between left and right hemisphere as has been suggested^46^. Furthermore, mosaicism is not necessarily evenly distributed within the same organ, so the random site of the biopsy can also affect the observed level of mosaicism. For this reason, it is hard to draw any firm conclusions on mosaic distribution and levels based on the results from the ddPCR experiments, but we report that all investigated tissues, representing all three germ layers, harbor the *APP* triplication. Single cell analysis with RNA profiling could provide more information on what cell types harbor the *APP* triplication, which could elucidate the timing of the mutation-event during embryogenesis. The mosaic state in subject II:2 implies a postzygotic, *de novo* event, and so her siblings are not at risk of carrying the derivative chromosome. However, children are at risk of having inherited the triplication, but the risk could not be estimated since we could not quantify the level of mosaicism in egg cells. Another limitation includes the few available tissues in the daughter, but a mosaic state in her would be unlikely since the triplication could only have been inherited in full heterozygous state.

## Conclusions

A mosaic *APP* triplication is the likely cause of EOAD in the mother. In addition, the increasing *APP* copy number in the fully heterozygous daughter leads to anticipation with an earlier age at onset and more aggressive disease progression. SVs and CGRs may be underdiagnosed in genetic disease and introduction of GS into the clinical setting will provide a tool for an increased identification of genetic neurodegenerative disorders (NDDs).

## Materials and methods

### Copy number analysis

Array comparative genome hybridization (array-CGH) was performed on blood DNA from both subjects as previously described^47^. DNA from two unrelated heterozygous carriers of *APP* duplications, from Finland and Sweden, were included in the analysis^32,47^.

### Tissue sampling and DNA extraction

Both subjects were ascertained at the Memory Clinic, Karolinska University Hospital. DNA extraction was performed using the DNeasy Blood and Tissue kit (Qiagen, Hilden, DE) from various tissues, including blood and another forty-three postmortem tissues in the mother (see Supplementary Table S2). No egg cells were available for genetic analysis. Tissue homogenization was achieved in ATL buffer using the TissueLyser LT with 40 s of vibration at 30 Hz. Samples that did not homogenize completely were additionally run in the TissueLyzer for another 40s and treated with proteinase-K overnight. DNA concentration was quantified using the Qubit^®^ dsDNA BR assay kit and the Qubit™ fluorometer from ThermoFisher Scientific (Waltham, Mass, USA).

### Brain tissue preparations

Material for histological staining was collected from the formaldehyde fixed right-sided brain regions according to a protocol for neurodegenerative disorders. In addition, frozen tissue from the left-side including all four brain lobes, anterior and posterior parts of the hippocampus, amygdala, basal ganglia, thalamus, midbrain, pons, medulla oblongata and cerebellum were collected in subject II:2. For subject III:3, frozen tissue from left cerebellum was collected for DNA extraction. See supplementary Table S2 for a detailed list over all tissues, including all frozen left-side brain regions, that were collected in subject II:2. Five µm thick paraffin sections were stained with haematoxylin and eosin, Luxol fast blue and Bielschowsky silver staining. Congo red was used for staining of amyloid. For subject III:3 immunostainings on selected regions were made with antibodies against hyperphosphorylated tau (Thermofisher) and against different epitopes of beta-amyloid (1–40 and 1–42, both Merck Millipore).

### Sequencing and bioinformatic analysis

DNA libraries were prepared with NeoPrep™ and GS was performed with HiSeq x Ten (Illumina instruments, via National genome infrastructure in Stockholm, Sweden) using 150 bp reads at a 20x coverage in 92.6% (mother) and 90.5% (daughter). BWA mem 2:2.2.1 was used as aligner. We used the MIP DNA pipeline v12.1.0 (https://github.com/Clinical-Genomics/MIP) for processing which includes the structural variant callers CNVnator v0.4.1 and TIDDIT v3.6.0^48,49^. An in-house analysis software called Scout was used for analysis of variants (https://github.com/Clinical-Genomics/scout) and genome data was visualized in Integrative Genomics Viewer (IGV, Broad Institute) using genome build GRCh37/hg19^50^. Breakpoint analysis was performed in University of California Santa Cruz Genomics Institute (UCSC) genome browser using genome build GRCh37/hg19 and with the human BLAT Search^51,52^. The clinically used neurodegeneration gene panel v8, including 138 genes, was used to exclude any disease-causing variant SNV or STR (see Supplementary Fig. S3). Sanger sequencing was performed as previously described^47^.

### ddPCR optimization and experiments

Primers and probes flanking the two unique BPJ sequences, obtained from the GS, were designed using the primer3plus tool (https://www.primer3plus.com/). Two assays were ordered from Integrated DNA Technologies™ (IDT, USA) and included primers and internal probes designed to cover the two unique BPJ sequences (see Supplementary Fig. S4). BPJ1; Forward: TCTTAGTCTGACTAAAGGTTTGTCA, Reverse: GGGAGGTGTGAAGGGGAAAA, (135 bp). Internal probe 1: SUN/ AACTAACTTGACTAACTTCTAGACTGAGG /BHQ. BPJ2; Forward: CCTGGATGTGAGACGTGGAG, Reverse: GTTCTCACCGCTGCTATGGA. Internal probe 2: HEX/TTGAAGCCACCGGGTGCGAC/BHQ (129 bp). Each amplicon and BPJ was confirmed by Sanger sequencing (see Supplementary Fig. S4). ddPCR assay optimization was conducted by gradient PCR (55.7-to-62 °C) in a Bio-Rad CFX96 PCR system (Bio-Rad, Hercules, CA, USA) according to manufacturer´s instructions. For this purpose, positive blood DNA from subject III:3 was used with the Bio-Rad ddPCR Supermix for Probes (No dUTP). Optimization and selection of the best primer/probe combination for ddPCR was performed according to manufacturer´s instructions. Optimal separation was achieved at 58.2 °C and 59.7 °C, with an average annealing temperature of 59 °C adopted in further ddPCR experiments. Since the BPJ2 assay displayed superior cluster separation compared to BPJ1 assay, BPJ2 assay was used in subsequent experiments.

Experiments, to define Limit of Detection (LOD) and Limit of Quantification (LOQ), were performed using serially diluted DNA from subject II:2 (31.7 ng/µL) in either water or triplication negative control (32.5 ng/µL) (not shown). For the LOQ experiment, 9 tubes with decreasing DNA concentration from subject II:2 and increasing triplication negative control DNA concentration were used. In addition, three non-DNA controls (H_2_O) were included as negative template controls (NTCs). For the LOD experiment, subject II:2 DNA was serially diluted by adding an increasing amount of H_2_O. The LOQ value was calculated by taking the SD for the three NTCs* 10 + 10 SD (~ 2.6 copies/µL) and the LOD value was calculated by taking the SD for the three most diluted wells*3 + 3SD (~ 0.95 copies/µL). In all experiments, the RPP30 assay from Bio-Rad was utilized as the human genome copy number reference for a diploid genome. Droplets were automatically generated in the QX200 Automated Droplet Generator (Bio-Rad, Hercules, CA, USA). For this, PCR mixtures were emulsified into 1nl droplets, generating 20,000 droplets per sample. The DG8 Droplet Generator cartridges were used containing 20 µl of the reaction mixture and 70 µl of QX200™ Droplet Generation Oil for probes. PCR reactions were further cycled as indicated above and samples were assayed in duplicates following optimization. To ensure accuracy, multiple negative control reactions, consisting of water mixed with the PCR reaction buffer, were included on each plate. Droplet fluorescence was quantified using the QX200 Droplet Reader, and data was managed in the QuantaSoftPro/ QX Manager Standard Edition software version 1.2.345 software (Bio-Rad, Hercules, CA, USA) and thresholds for target positive and negative droplets and negative controls (water and PCR buffer) were done manually according to manufacturer´s user manual. See also supplementary figure S8 for example and comparison of manual vs. automatic thresholding in selected tissues. The mean concentration (copies/µL) for BPJ2 and RPP30 respectively was calculated and the variant allele frequency (VAF) was computed using this formula: VAF = (Mean concentration of positive droplets for BPJ2/ Mean concentration of positive droplets for Reference RPP30​)*100 for each DNA. Mosaicism was calculated by taking the VAF value *2, reflecting the percentage of cells with heterozygous presence of the *APP* triplication state in each cell.

## Electronic supplementary material

Below is the link to the electronic supplementary material.

Supplementary Material 1

Supplementary Material 2

## Data Availability

Most of the data generated or analyzed during this study are included in this published article and its supplementary information files. Further used and/or analyzed data in the current study is available from the corresponding author upon reasonable request.

## Abbreviations

Aβ: amyloid-β
AD: Alzheimer disease
ADAD: autosomal dominant Alzheimer disease
APOE: apolipoprotein E
APP: amyloid-β precursor protein gene
Array-CGH: array comparative genome hybridization
BPJ: breakpoint junction
CAA: cerebral amyloid angiopathy
CGR: complex genomic rearrangement
CNV: copy number variants
CSF: cerebrospinal fluid
CT: computer tomography
ddPCR: digital droplet PCR
EOAD: early onset Alzheimer disease
ES: exome sequencing
FoSTeS: fork stalling and template switching
GS: genome sequencing
ICH: intracerebral hemorrhage
IGV: Integrative Genomics Viewer
Indel: insertion/deletion
LINE: long interspersed nuclear element
LOAD: late onset Alzheimer disease
LOD: limit of detection
LOF: loss of function
LOQ: limit of quantification
LTR: long terminal repeat
MMBIR: microhomology-mediated break-induced replication
MRI: magnetic resonance imaging
NDD: neurodegenerative disorder
NTC: negative template control
PD: Parkinson disease
PGT: preimplantation genetic testing
PSEN1: presenilin 1
PSEN2: presenilin 2
SNV: single nucleotide variant
SPECT: Single-Photon Emission Computerized Tomography
STR: short tandem repeat
SV: structural variant
VAF: variant allele frequency
