# Author Correction: A de novo, mosaic and complex chromosome 21 rearrangement causes *APP* triplication and familial autosomal dominant early onset Alzheimer disease

**DOI:** 10.1038/s41598-025-94140-9

**Published:** 2025-03-24

**Authors:** Emma Ehn, Jesper Eisfeldt, Jose M. Laffita-Mesa, Håkan Thonberg, Jacqueline Schoumans, Anne M. Portaankorva, Matti Viitanen, Anna Lindstrand, Inger Nennesmo, Caroline Graff

**Affiliations:** 1 Division for Neurogeriatrics, Centre for Alzheimer Research, Department of Neurobiology, Care Sciences and Society, Karolinska Institutet, Stockholm, Sweden; 2 Unit for Hereditary Dementias, Karolinska University Hospital Solna, Stockholm, Sweden; 3 Department for Molecular Medicine and Surgery, Karolinska Institutet, Stockholm, Sweden; 4 Department of Clinical Genetics and Genomics, Karolinska University Hospital Solna, Stockholm, Sweden; 5 Département de Médicine de Laboratoire et Pathologie, Centre Universitaire Hospitalier Vaudois (CHUV), Lausanne, Switzerland; 6 Faculty of Medicine, Clinical Neurosciences, University of Helsinki, Helsinki, Finland; 7 Division for Clinical Geriatrics, Department of Neurobiology, Care Sciences and Society, Karolinska Institutet, Huddinge, Sweden; 8 Department of Oncology-Pathology, Karolinska Institutet, Stockholm, Sweden; 9 Department of Pathology and Cancer Diagnostics, Karolinska University Hospital Solna, Stockholm, Sweden; 10 Department of Clinical Neuroscience, Karolinska Institutet, Stockholm, Sweden

Correction to: *Scientific Reports* 10.1038/s41598-025-86645-0, published online 6 January 2025

The original version of this Article contained an error in the spelling of the author Inger Nennesmo which was incorrectly given as Inger Nennesemo.

The original Article has been corrected.

