# Supplementary material for: A de novo, mosaic and complex chromosome 21 rearrangement causes APP triplication and familial autosomal dominant early onset Alzheimer disease
Source: Sci Rep. 2025 Jan 23;15:2912. doi: 10.1038/s41598-025-86645-0 (PMC11759332; doi:10.1038/s41598-025-86645-0)
Supplement: Supplementary file 2 — Supplementary Material 2 [file 41598_2025_86645_MOESM2_ESM.pdf]

“A *de novo*, mosaic and complex chromosome 21 rearrangement causes *APP* triplication and familial autosomal dominant early onset Alzheimer disease”

Scientific Reports

Emma Ehn, Jesper Eisfeldt, Jose M Laffita-Mesa, Håkan Thonberg, Jacqueline Schoumans, Anne M. Portaankorva, Matti Viitanen, Anna Lindstrand, Inger Nennesmo and Caroline Graff

Corresponding author: Emma Ehn, Karolinska Institutet, Division for Neurogeriatrics, Centre for Alzheimer Research, Department of Neurobiology, Care Sciences and Society, Stockholm, Sweden. Karolinska University Hospital Solna, Unit for Hereditary Dementias, Stockholm, Sweden
emma.ehn@ki.se

Supplementary Figure S1

Array comparative genome hybridization

Array-CGH included analysis of subject II:2 (pink line) and III:3 (black line) and two *APP* duplications from Finland and Sweden (1)(2). The size of the copy number increased region (1.06 Mb) is larger in subject II:2 and III:3 compared to the Finnish (blue line, 0,713 Mb) and Swedish (green line, 0,947 Mb) cases.

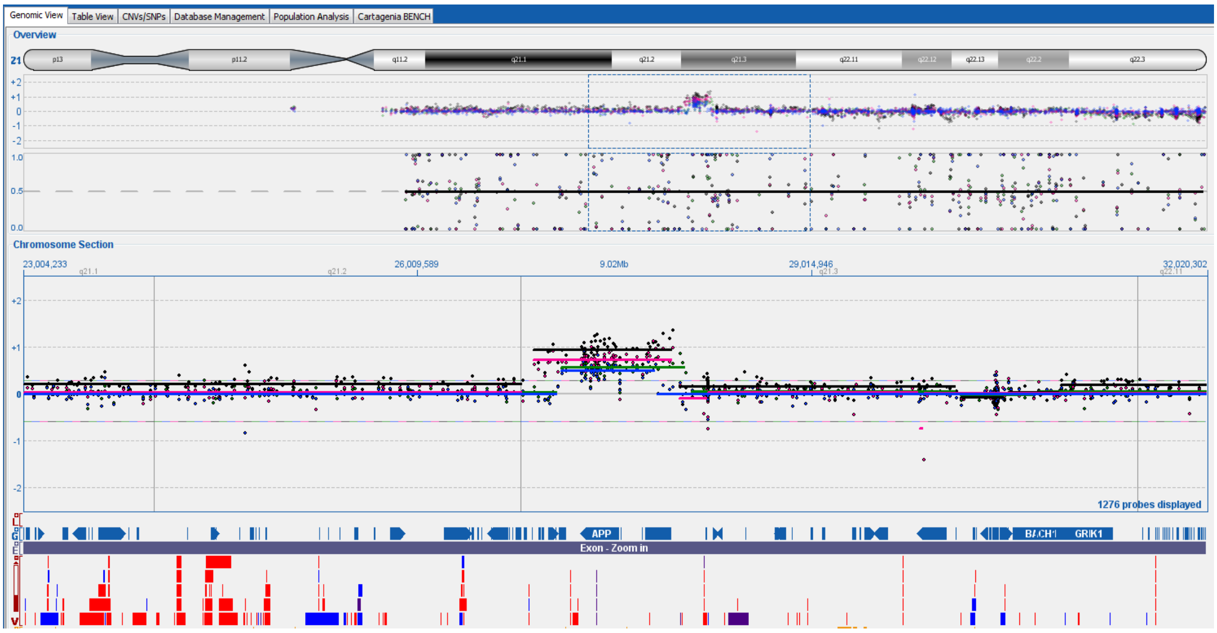

1. Rovelet-Lecrux A, Frebourg T, Tuominen H, Majamaa K, Campion D, Remes AM. APP locus duplication in a Finnish family with dementia and intracerebral haemorrhage. J Neurol Neurosurg Psychiatry. 2007;78(10):1158.

2. Thonberg H, Fallström M, Björkström J, Schoumans J, Nennesmo I, Graff C. Mutation screening of patients with Alzheimer disease identifies APP locus duplication in a Swedish patient. BMC Res Notes [Internet]. 2011;4(1):476. Available from: http://www.biomedcentral.com/1756-0500/4/476

Supplementary Figure S2

Short read GS results in IGV in subject III:3 (daughter)

Breakpoint 1

The informative reads are in blue (inverted compared to reference). Breakpoint 1 is depicted by the sharp increase in read depth (red arrow, read depth = grey bars in top panel).

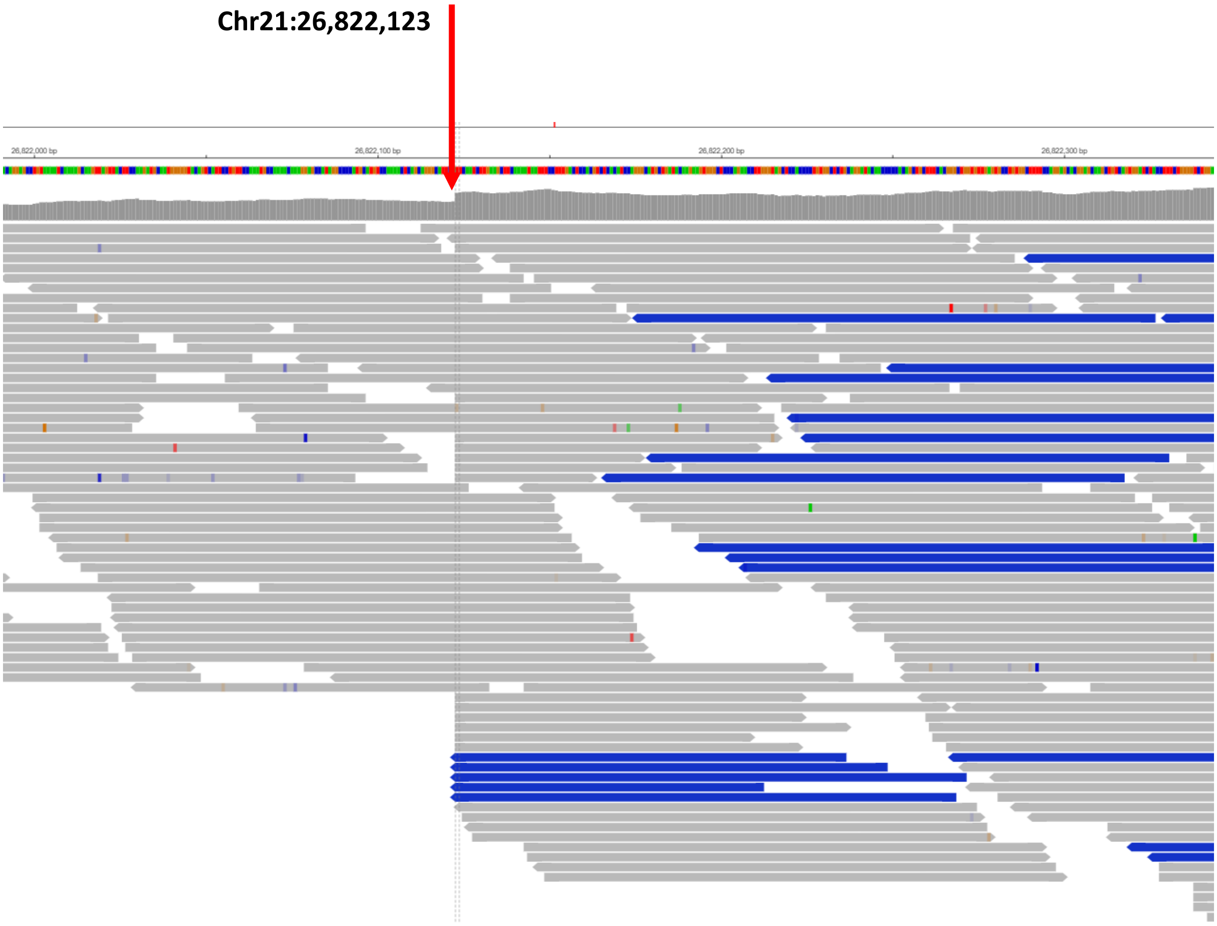

Breakpoint 2

Increase in read depth shows the position of breakpoint 2. In addition, this second breakpoint is within the first exon of the *MRPL39* gene, which is shown in the top panel.

Chr21: 26,979,728

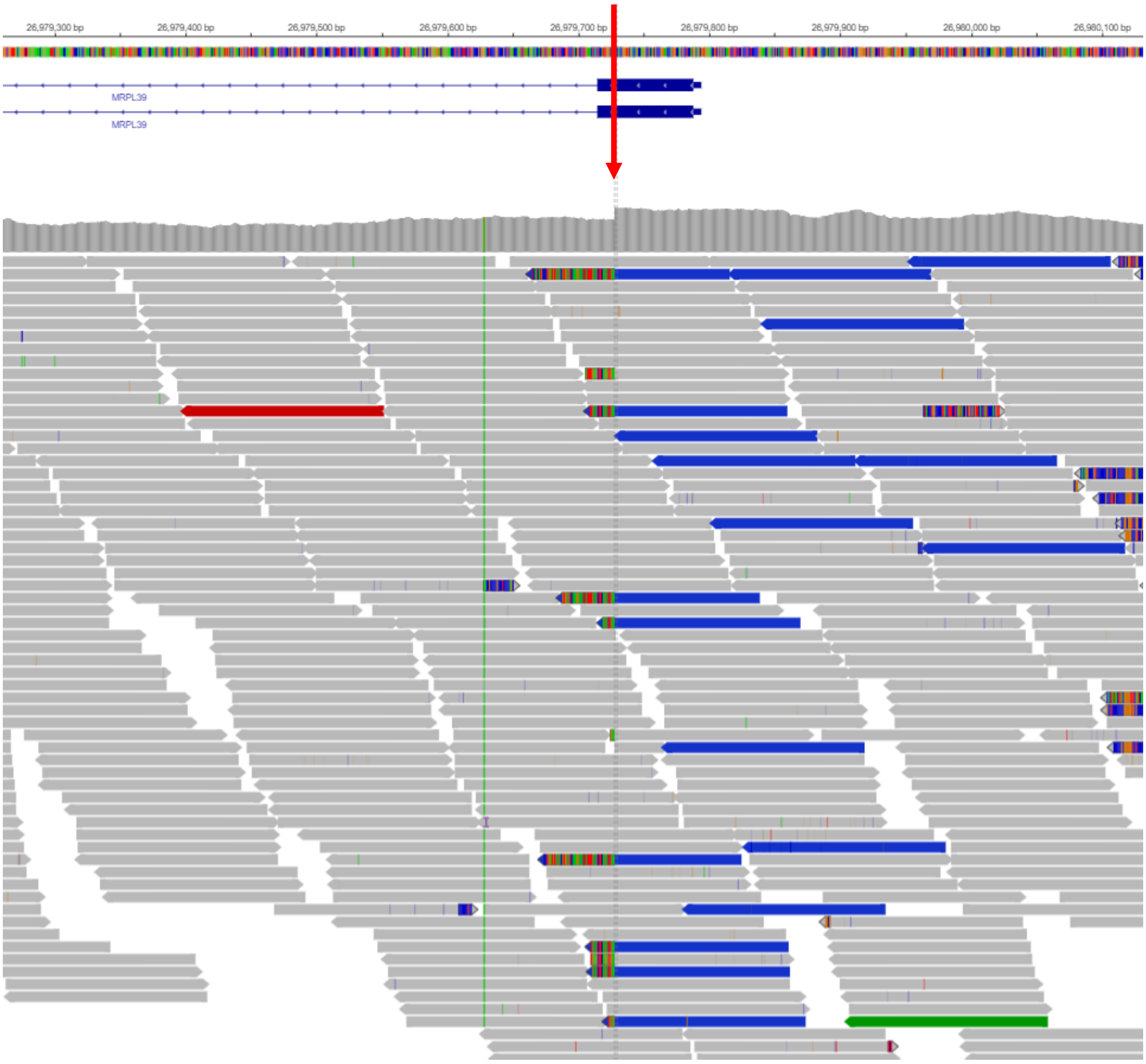

Breakpoint 3

The increase in read depth (red arrow) shows breakpoint 3. Informative (inverted) reads are in turquoise.

Chr21:27,977,994

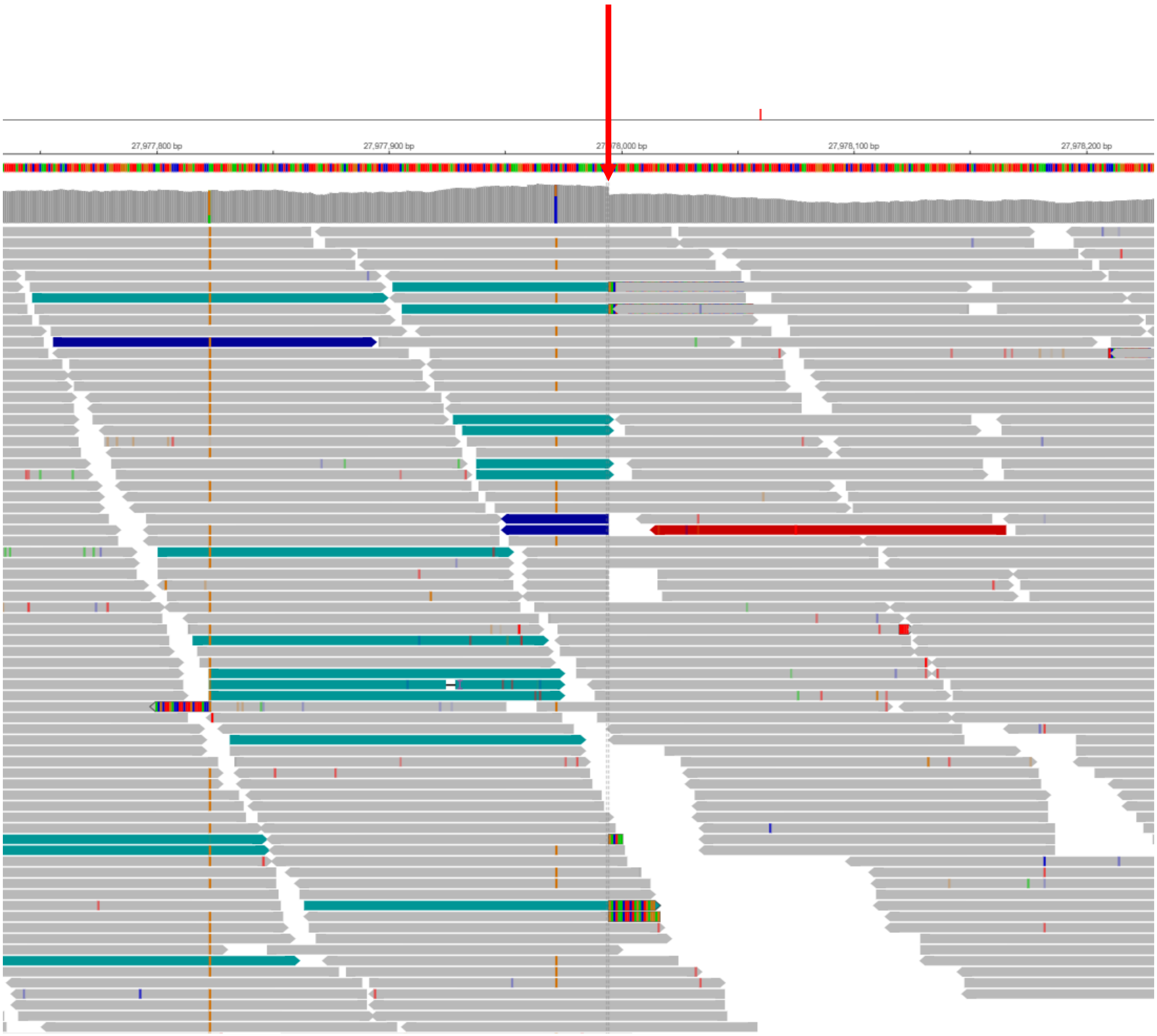

Templated insertion in breakpoint 3 (daughter)

A closer view of the same region as above (breakpoint 3) showing the templated insertion (GACTAA), shown in figure 5 in the paper.

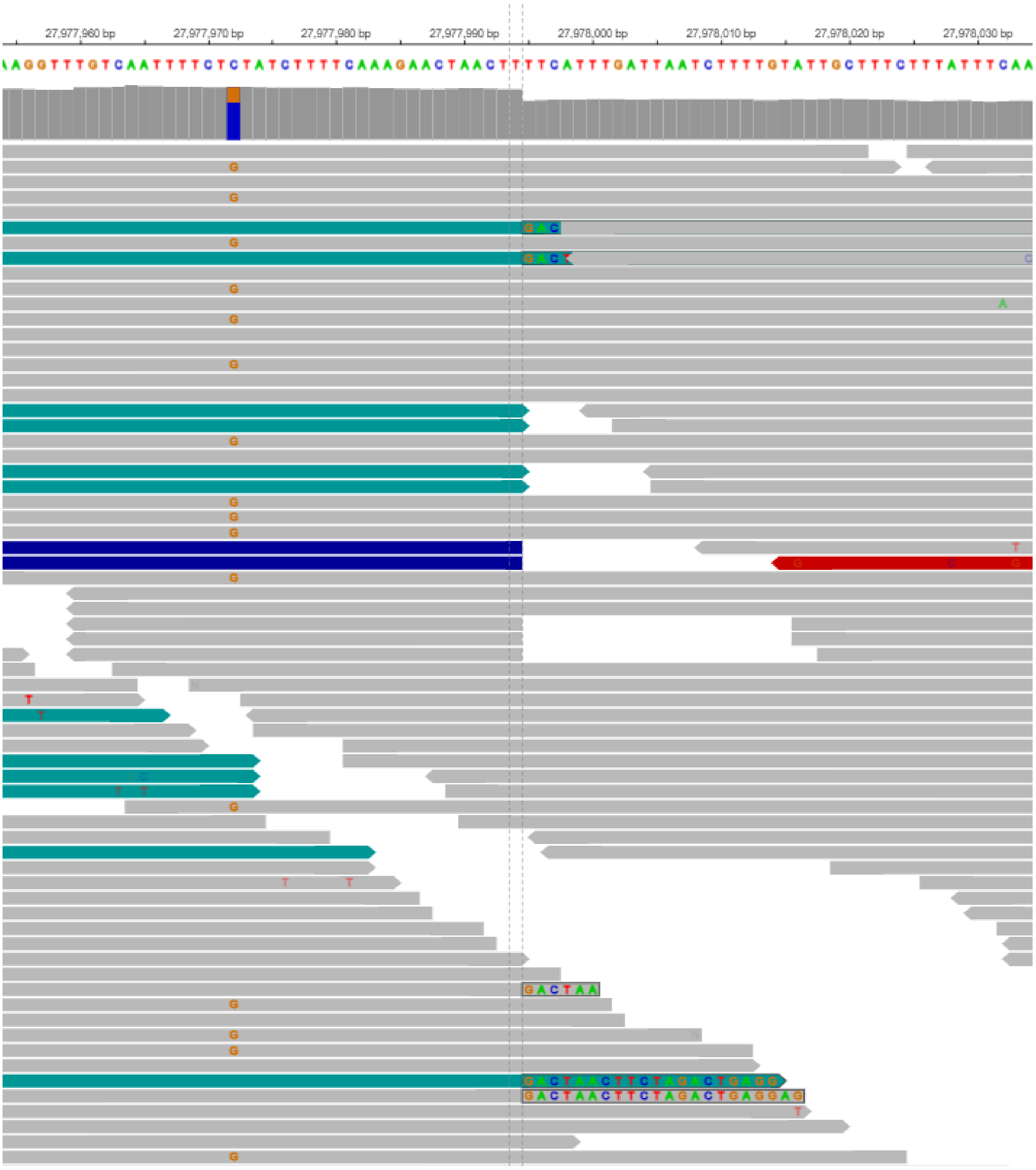

Breakpoint 4

Increase in read depth is depicted by the red arrow. Informative (inverted) reads are in turquoise.

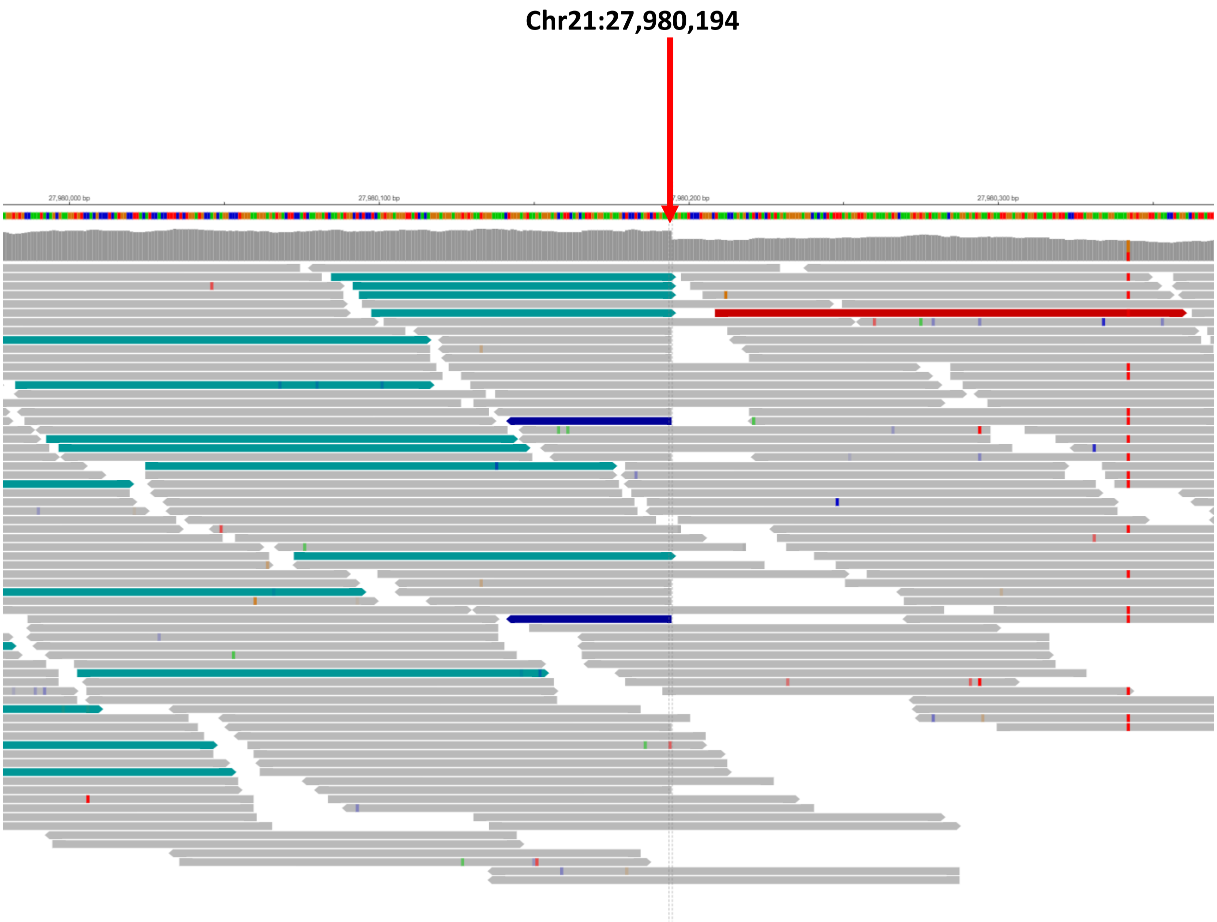

Short read GS results in IGV in subject II:2 (mother)

Breakpoint 1

IGV view in subject II:2 showing the same genomic positions as above. Only a couple of blue reads are present, indicating a mosaic state. In addition, the increase in read depth is not as distinct as in the full heterozygous state in subject III:3, further supporting mosaicism. The red arrow shows breakpoint 1.

Chr21: 26,822,123

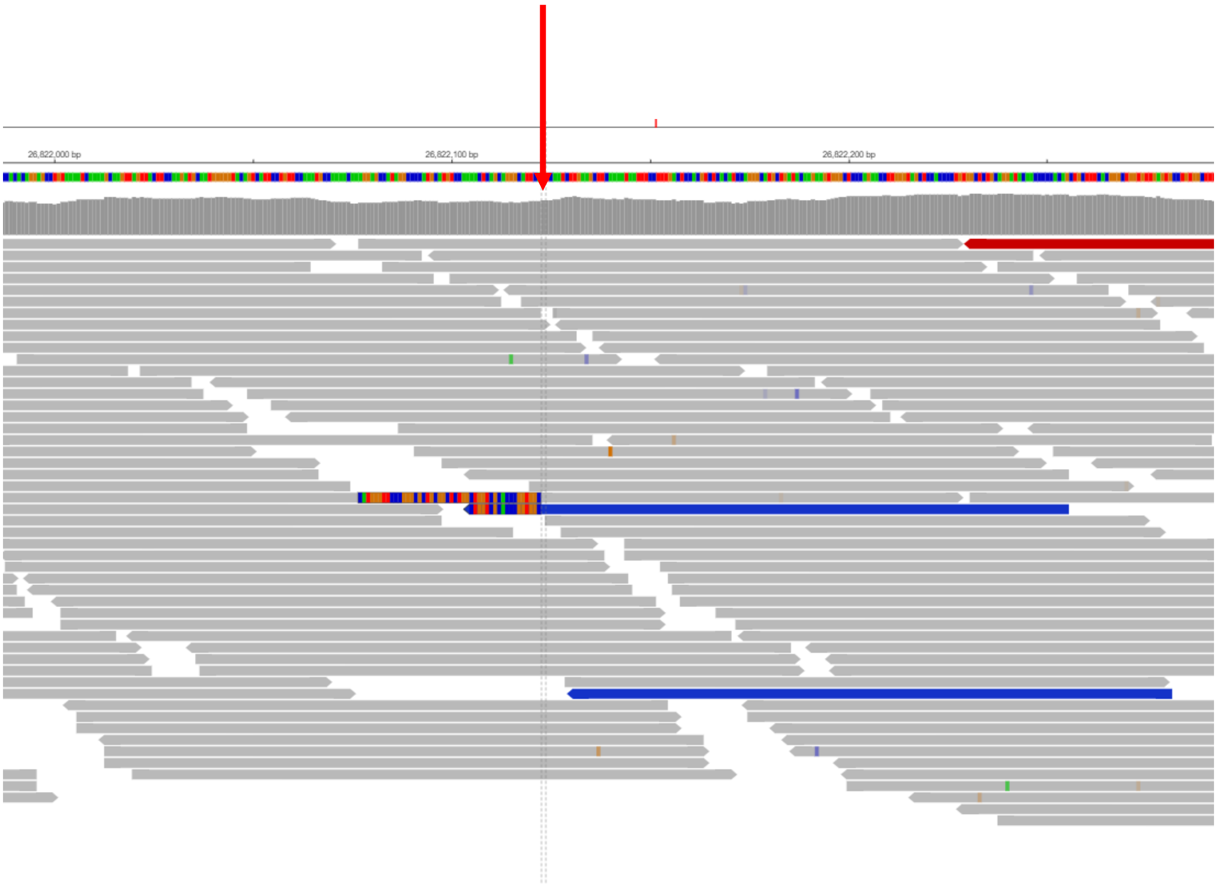

Breakpoint 2

Breakpoint 2, depicted by the red arrow, is within the first exon of the *MRPL39* gene shown in the top panel. Again, only a couple of reads (blue) indicate inverted orientation of the SV. Read depth increase is not as sharp as in subject III:3.

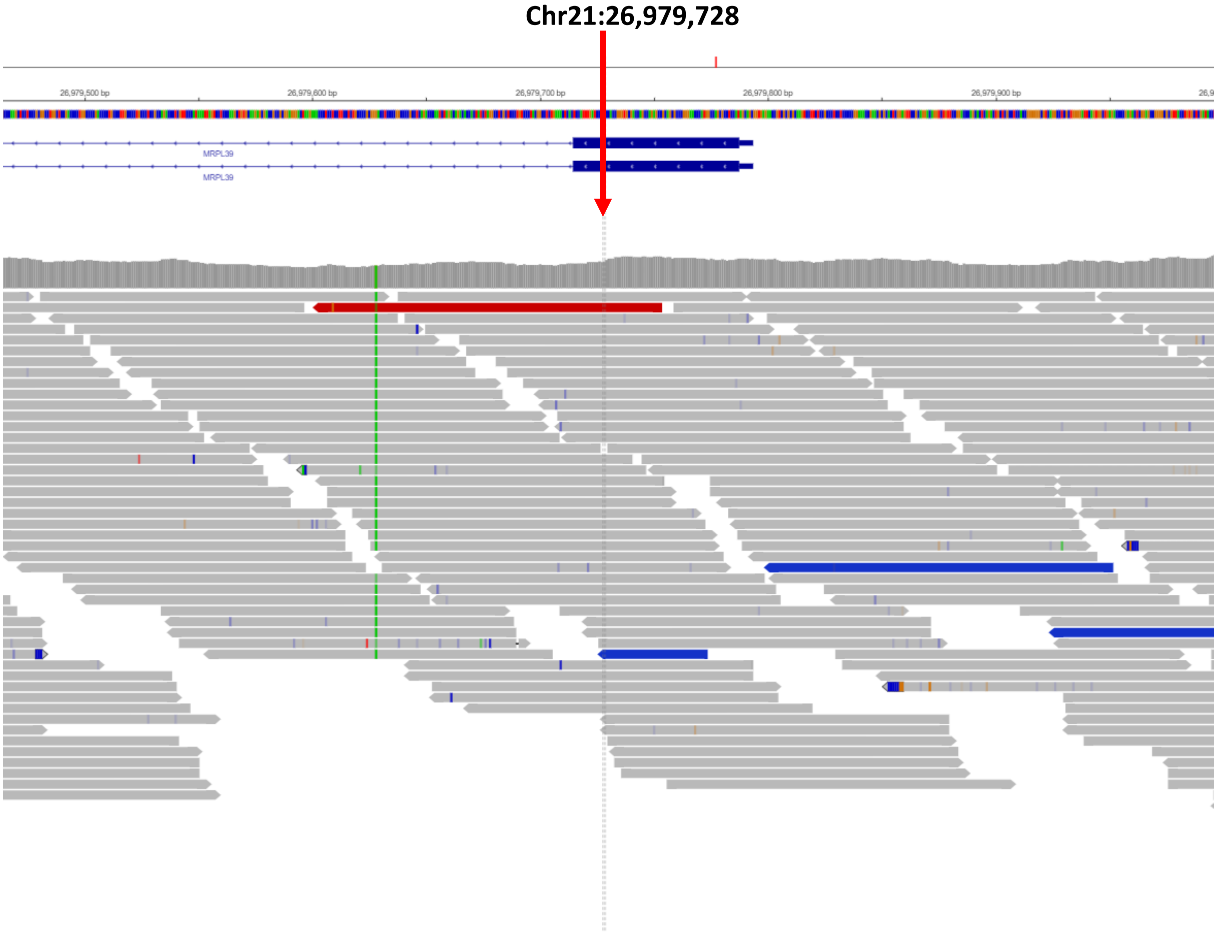

Breakpoint 3

The red arrow depicts breakpoint 3, but only a few informative reads (turquoise) are present and there is no apparent read depth increase over the breakpoint.

Chr21:27,977,994

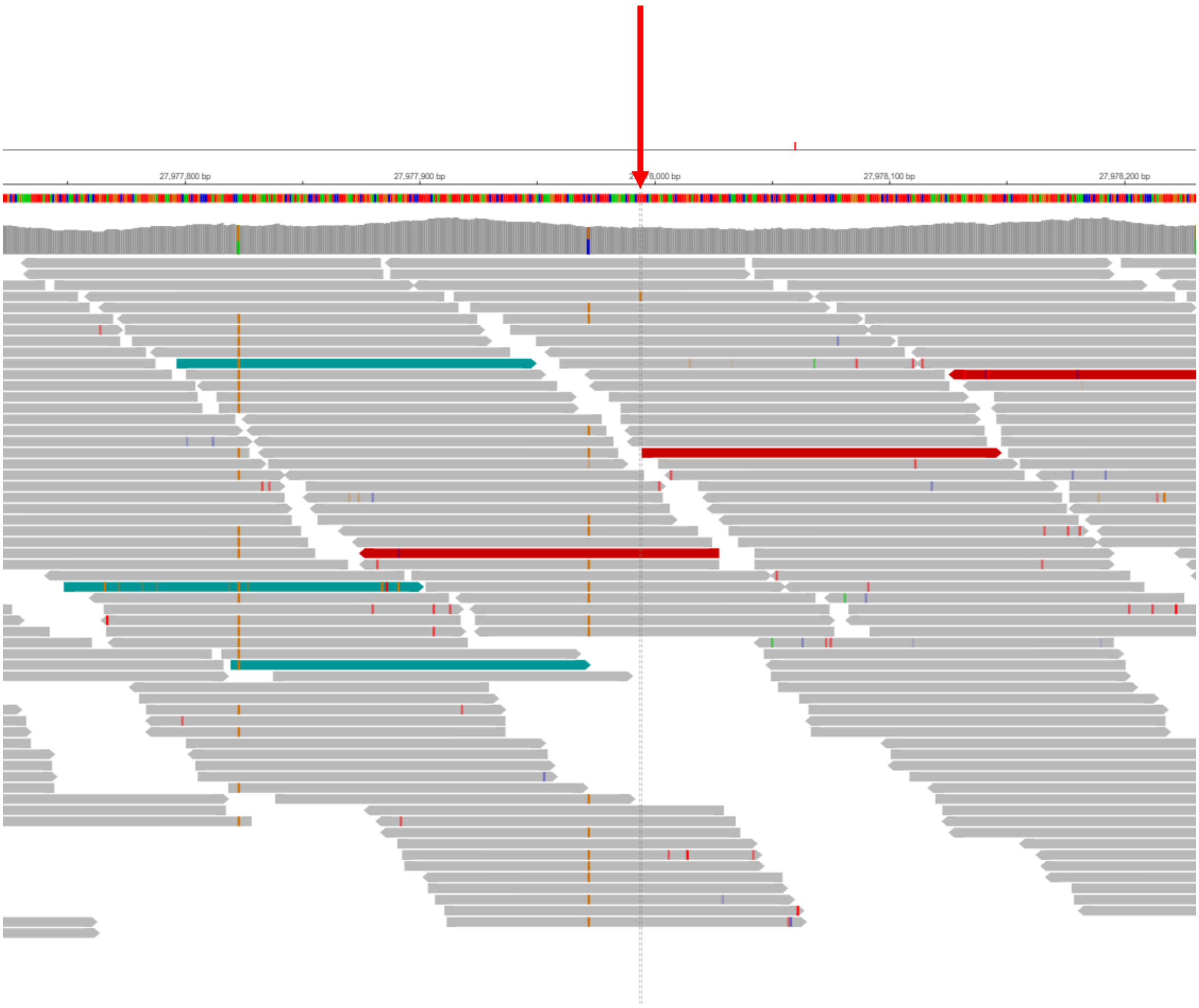

Breakpoint 4

In breakpoint 4, depicted by red arrow, there is no sharp read depth increase and there are only a couple of informative (turquoise) reads.

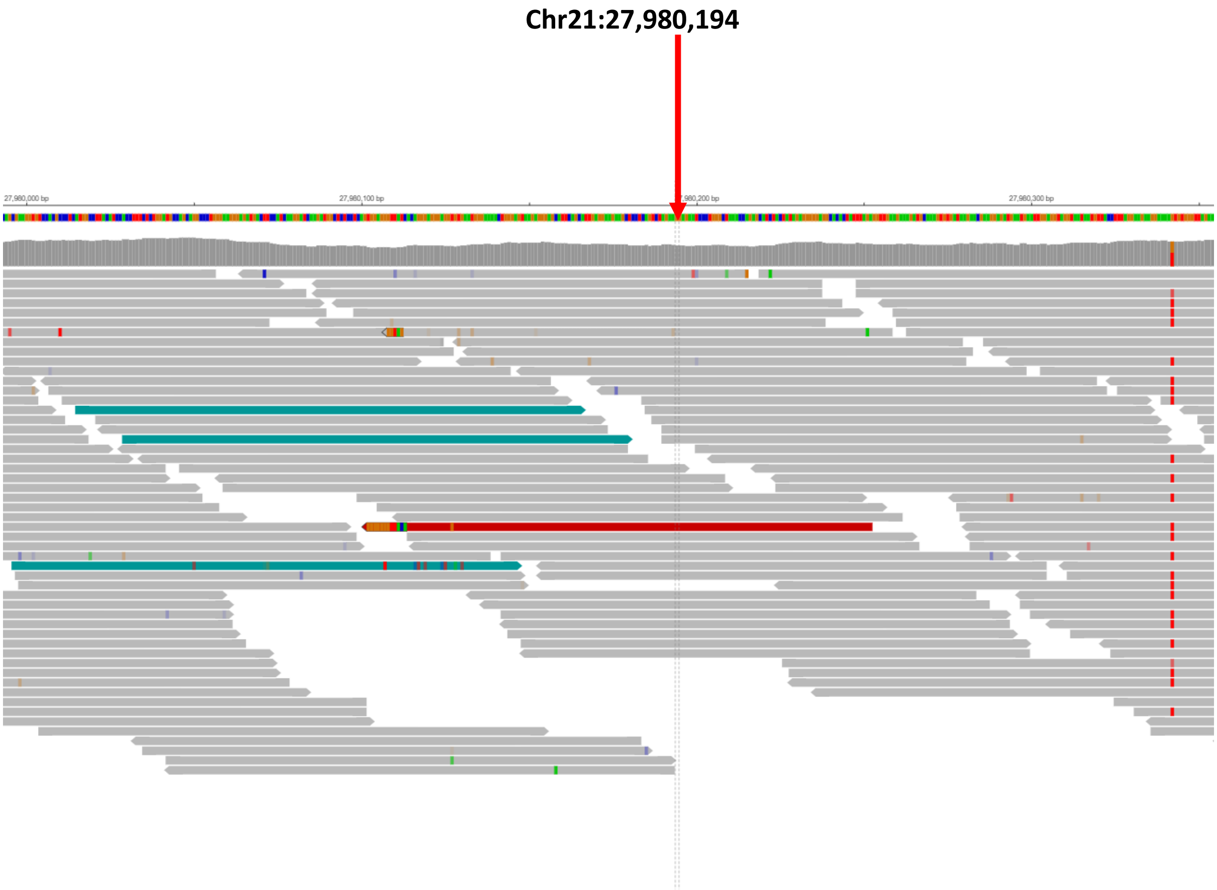

Supplementary Figure S3

Neurodegeneration panel v 8 (138 genes)

ABCD1, AFG3L2, ALS2, ANG, ANXA11, APP, AR, ARSA, ATN1, ATP13A2, ATP1A3, ATP7B, ATXN1, ATXN10, ATXN2, ATXN3, ATXN7, ATXN8OS, AUH, C19orf12, C9orf72, CACNA1A, CACNA1G, CCNF, CHCHD10, CHCHD2, CHMP2B, CLCN2, CLN6, COASY, COL4A1, COL4A2, CP,CSF1R, CTSA, CTSF, CYLD, CYP27A1, CYP7B1, DARS2, DCTN1, DNAJC5, DNAJC6, DNMT1, EIF2B1, EIF2B2, EIF2B3, EIF2B4, EIF2B5, ELOVL4, EPM2A, ERBB4, FBXO7, FIG4, FTL, FUS, FXN, GBA, GCH1, GFAP, GLA, GRN, GSN, HEXA, HEXB, HNRNPA1, HTRA1, HTT, ITM2B, JAM2, JPH3, KCNC3, KCND3, KIF5A, LAMB1, LRRK2, LYST, MAPT, MATR3, NHLRC1, MYORG, NIPA1, NOP56, NOTCH3, NPC1, NPC2, OPA3, OPTN, PANK2, PARK7, PDGFB, PDGFRB, PFN1, PINK1, PLA2G6, PLD3, PPP2R2B, PRKN, PRKRA, PRNP, ~~PRPH~~, PSEN1, PSEN2, RAB39B, RFC1, RNF216, SETX, SIGMAR1, SLC20A2, SLC30A10, SNCA, SOD1, ~~SORL1~~, SPAST, SPG11, SPR, STUB1, SQSTM1, SYNJ1, TARDBP, TBK1, TBP, TMEM240, TREM2, TREX1, TTC19, TTR, TUBA4A, TUBB4A, TYROBP, UBQLN2, VAPB, VCP, WDR45, VPS13A, VPS35, XPR1

Whereof 18 repeat expansion genes: AR, ATN1, ATXN1, ATXN10, ATXN2, ATXN3, ATXN7, ATXN8OS, C9orf72, CACNA1A, FXN, HTT, JPH3, NIPA1, NOP56, PP2R2B, RFC1, TBP

Genes not included in gene panel versions in use when loading this case (clinical list):'PRPH', 'SORL1'

Supplementary Figure S4

Fig 1a Primer assays

BPJ1

SnapGene summary showing the set of primers (purple) and internal probe (blue) for BPJ1. The unique BPJ1 sequence is shown in **yellow.**

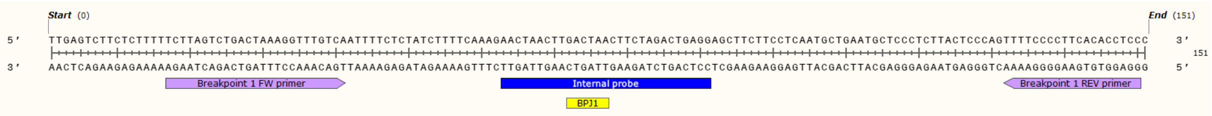

BPJ2

SnapGene summary showing the set of primers (pink) and internal probe (green) for BPJ2. The blunt BPJ2 is depicted by an arrow.

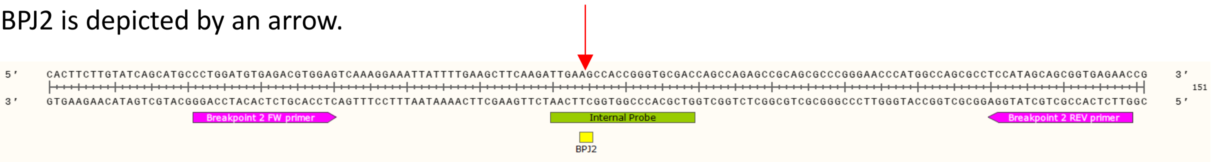

Fig 1b Sanger sequencing

BPJ1

Sequence from the BPJ1 forward (FW) sequencing in subject III:3. The unique BPJ1 sequence is highlighted in blue. The reverse (REV) primer sequencing did not yield satisfactory sequence covering BPJ1.

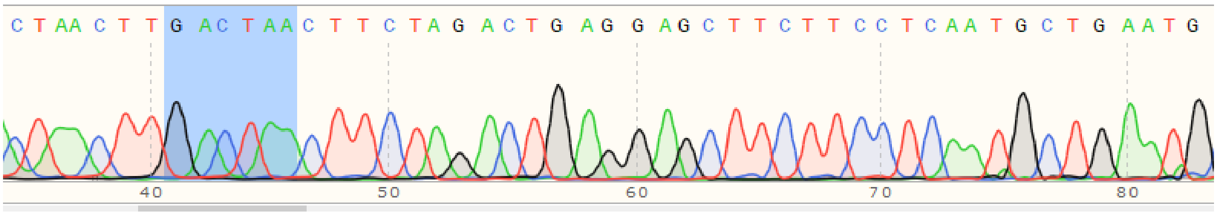

BPJ2

Sequence from the BPJ2 FW sequencing in subject III:3. The blunt BPJ2 is depicted by an arrow.

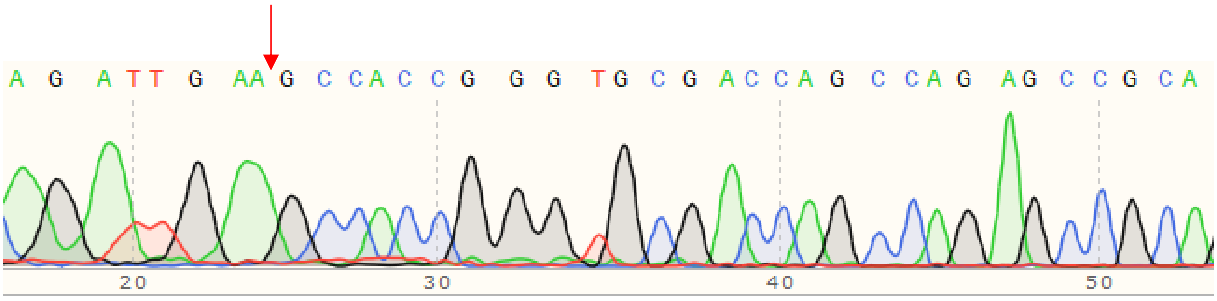

Sequence from the BPJ2 REV sequencing in subject III:3. The blunt BPJ2 is depicted by an arrow.

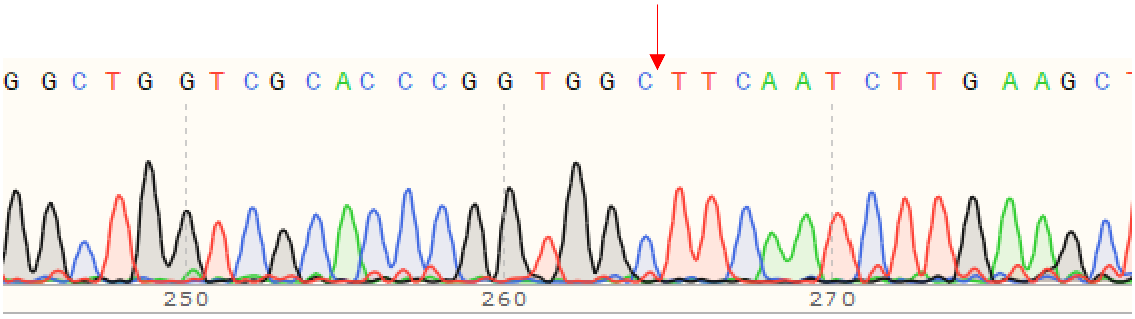
G G C T G G T C G C A C C C G G T G G C T T C A A T C T T G A A G C T
250
260
270

Supplementary Figure S5

Analysis of breakpoints in UCSC

Breakpoint 1

Breakpoint 1 (chr21: 26,822,123) is in a long terminal repeat (LTR). The red arrow depicts the breakpoint 1 position.

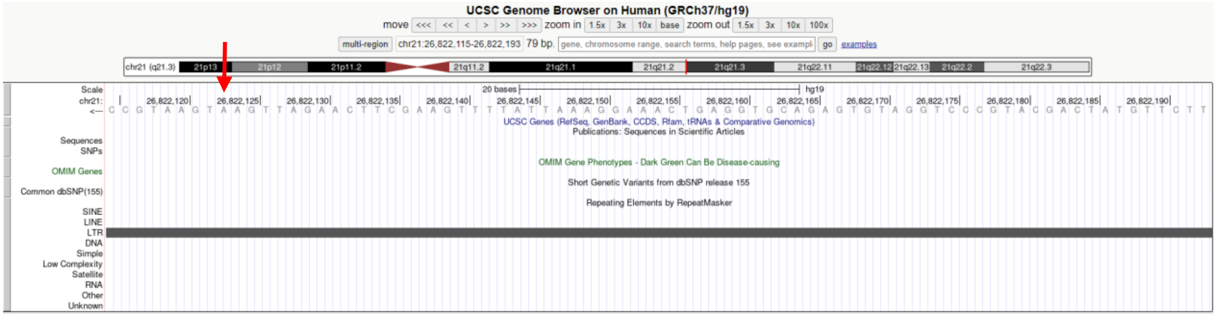

Breakpoint 2

Breakpoint 2 (chr21: 26,979,728) is in the middle of the first exon in the *MRPL39* gene. The red arrow depicts the breakpoint 2 position.

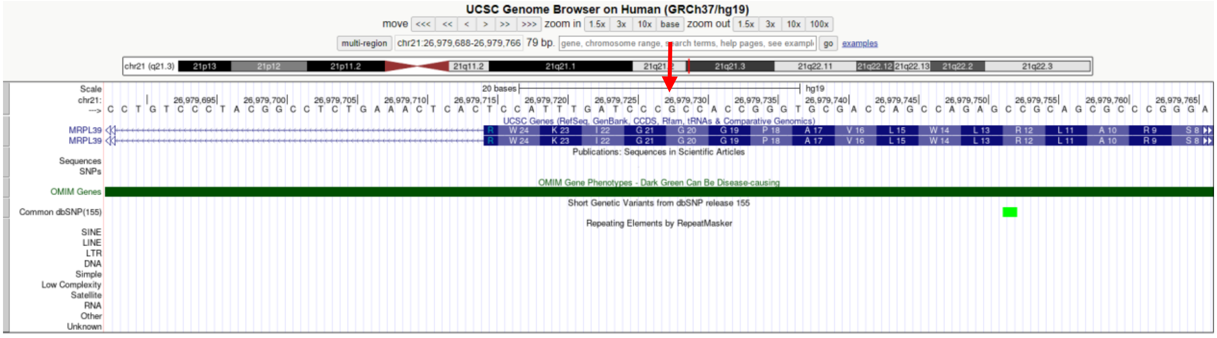

Breakpoint 3

Breakpoint 3 (chr21: 27,977,994) is within a long interspersed nuclear element (LINE). The red arrow depicts the breakpoint 3 position.

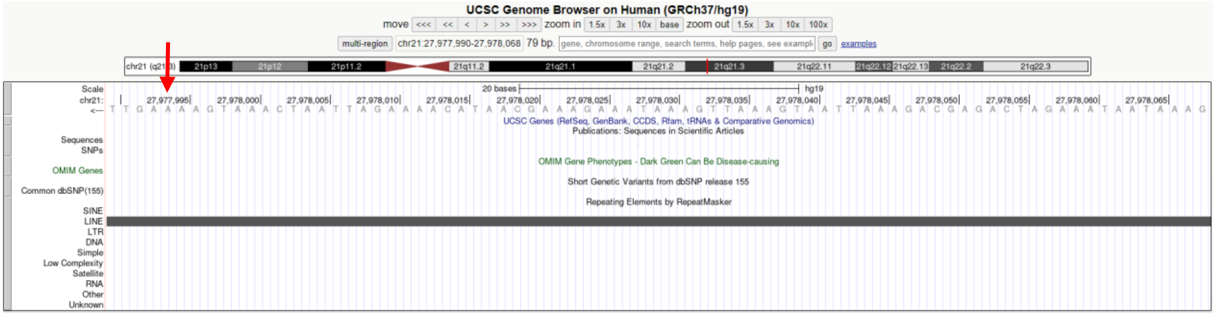

Breakpoint 4

Breakpoint 4 (chr21: 27,980,194) is also within an LTR (LTR16A, family: ERVL). The red arrow depicts the breakpoint 4 position.

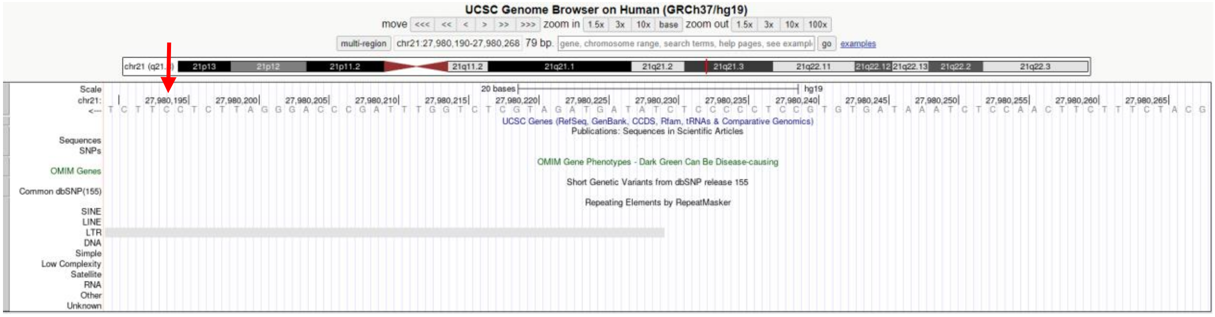

Supplementary Figure S6

Mosaicism left and right side in subject II:2

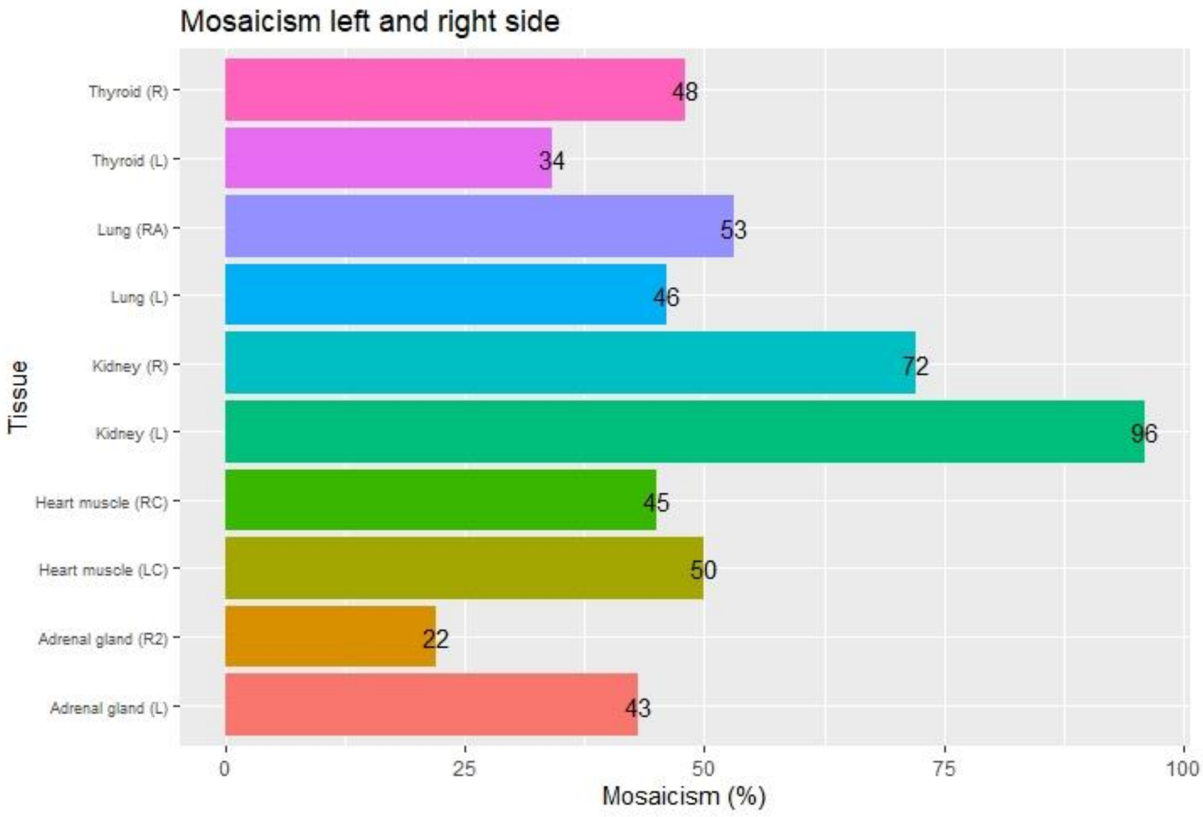

Mosaic levels in tissues sampled from left and right part of the body. R= right, L= left, RA= right apical, RC= right chamber, LC= left chamber

Supplementary Figure S7

Mosaicism from different part of the same tissue in subject II:2

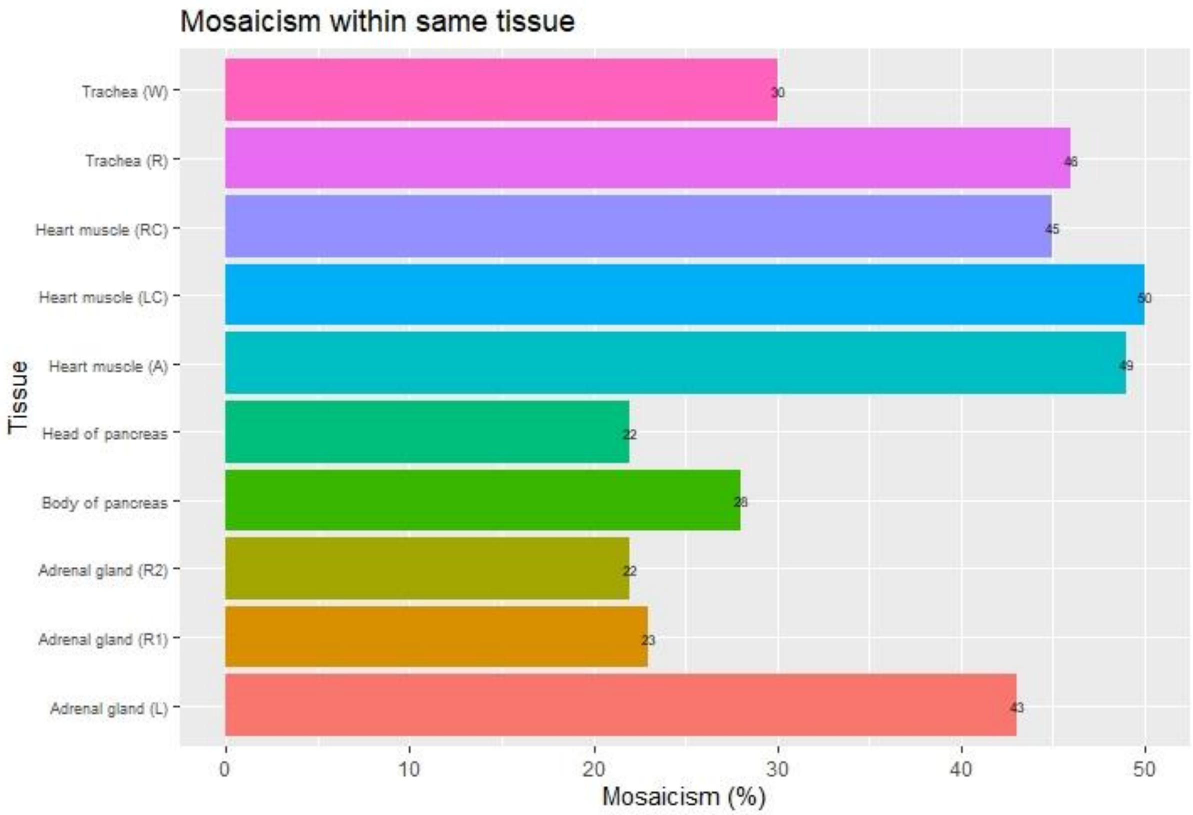

Mosaic levels in tissues sampled from different parts of the same tissue. W= white, R= right, RC= right chamber, LC= left chamber, A= atrium

Supplementary Figure S8

ddPCR results in subject III:3 (cerebellum) and selected tissue from subject II:2 with different mosaic levels (cerebellum 32% mosaicism, temporal pole 20% mosaicism, liver right 44% and left kidney 96% mosaicism) illustrated in 1D and 2D plots.

Plots comparing automatic and manual thresholding in different tissues.

ddPCR results in subject III:3 (daughter)

Cerebellum

1D plot breakpoint 2 assay

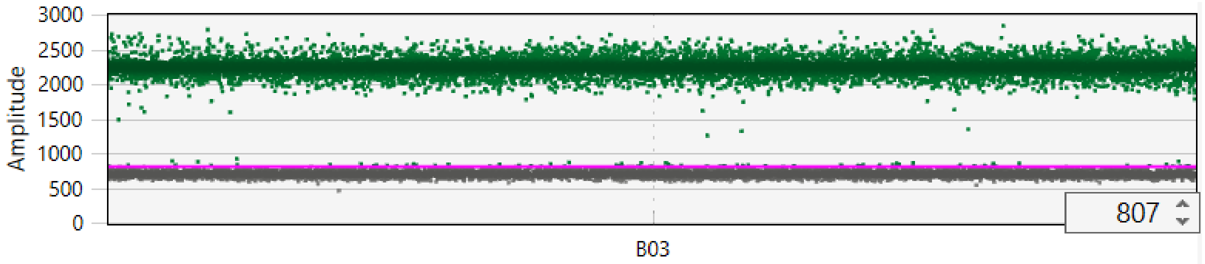

2D plot breakpoint 2 assay

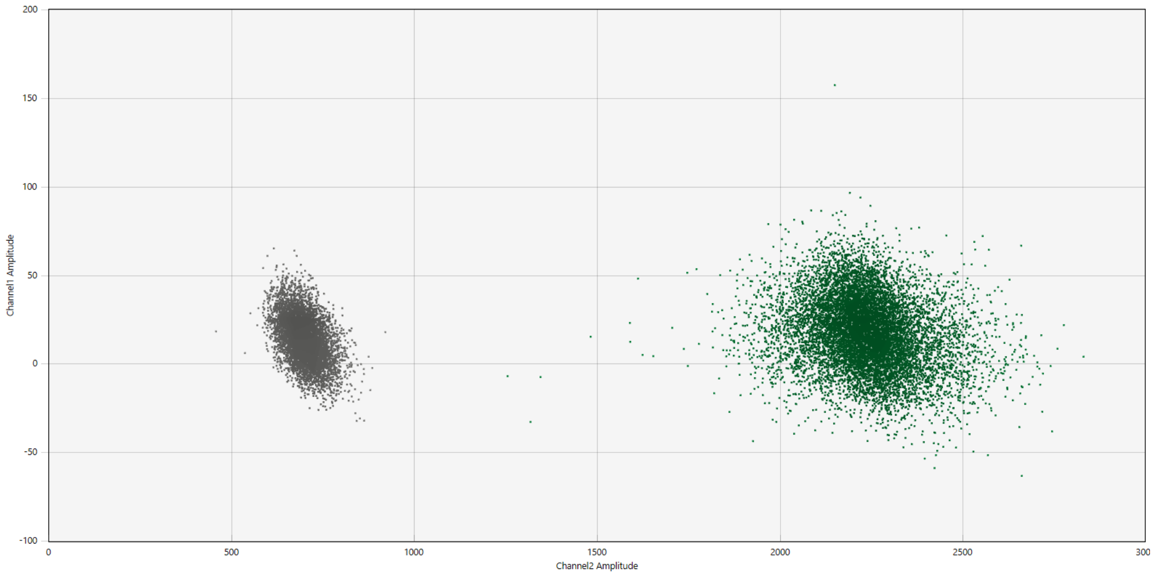

1D plot RPP30 assay (reference)

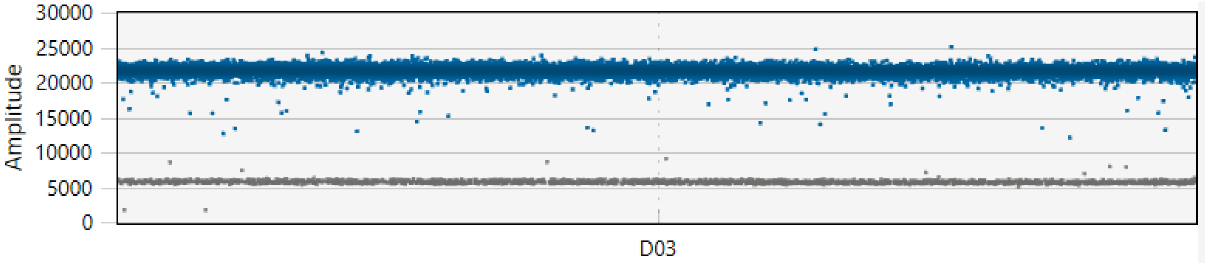

2D plot RPP30 assay (reference)

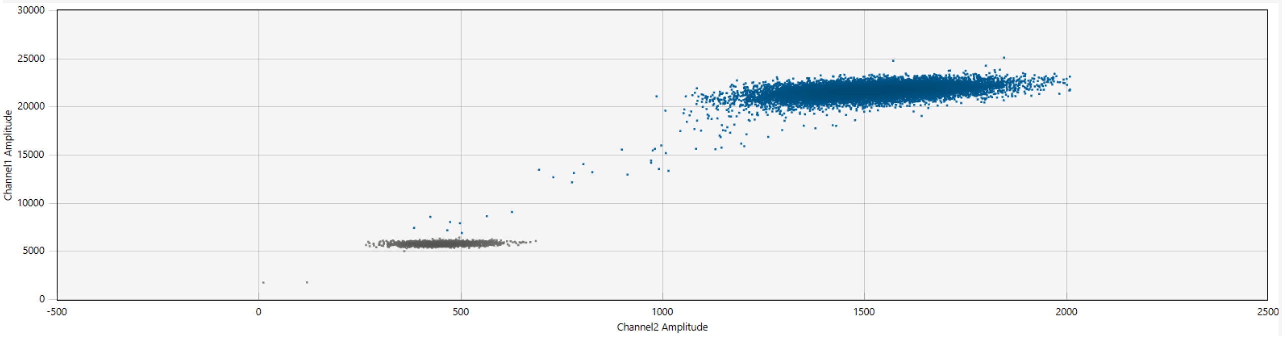

ddPCR results in subject II:2 (mother)

Cerebellum (32% mosaicism)

1D plot breakpoint 2 assay

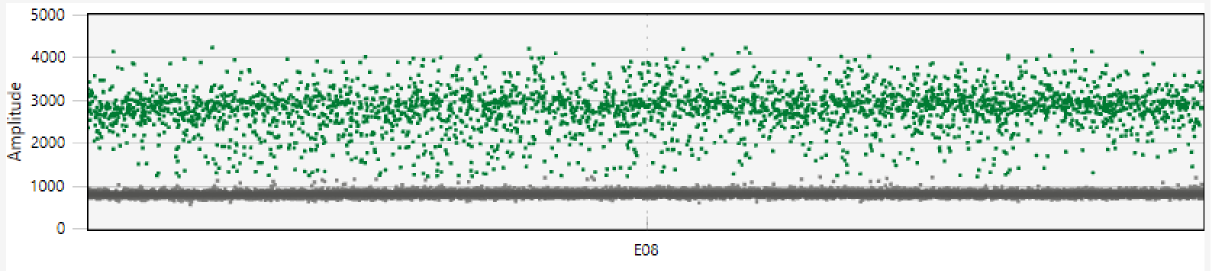

2D plot breakpoint 2 assay

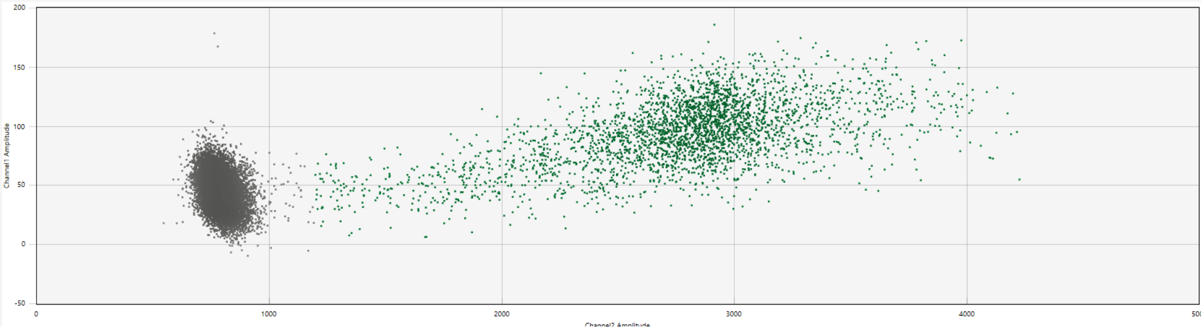

1D plot RPP30 assay (reference)

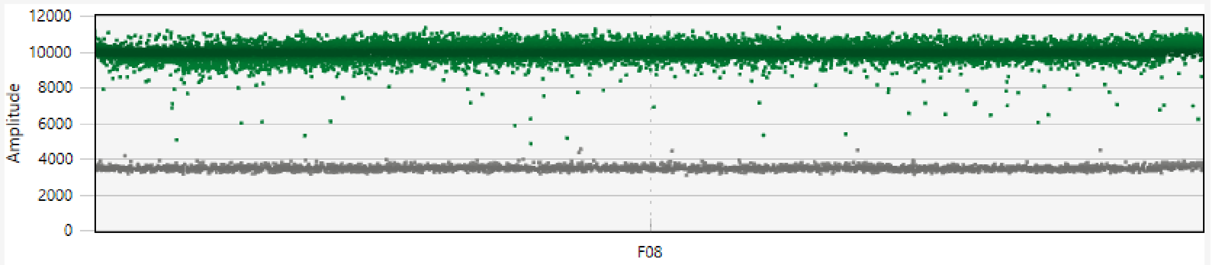

2D plot RPP30 assay (reference)

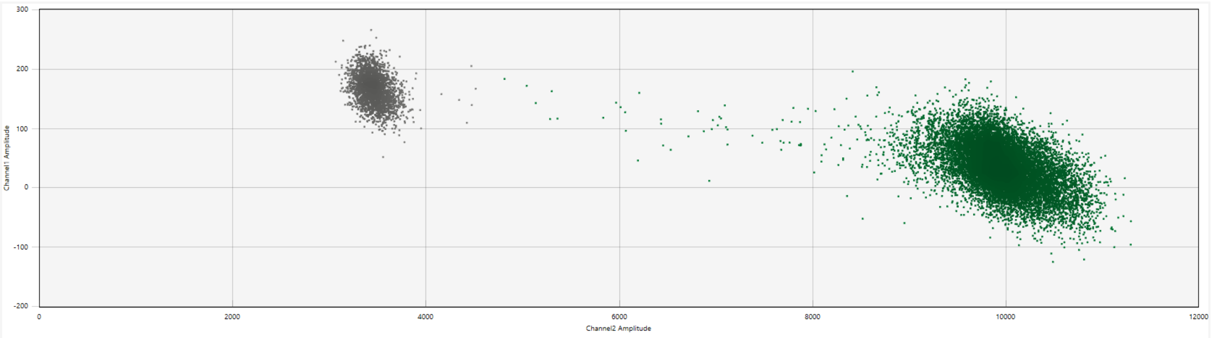

Results from three tissues with different mosaic levels: temporal pole (20%), liver right (44%) and left kidney (96%).

1D plot breakpoint 2 assay

Temporal pole	Liver (R)	Kidney (L)

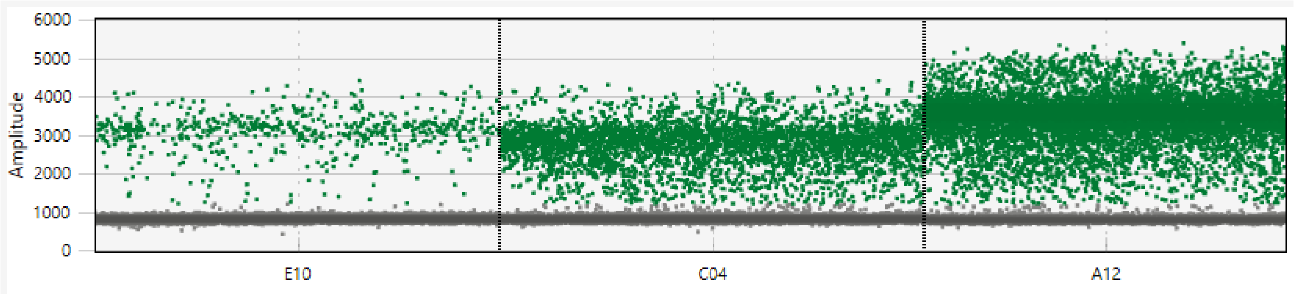

1D plot RPP30 assay (reference)

Temporal pole	Liver (R)	Kidney (L)

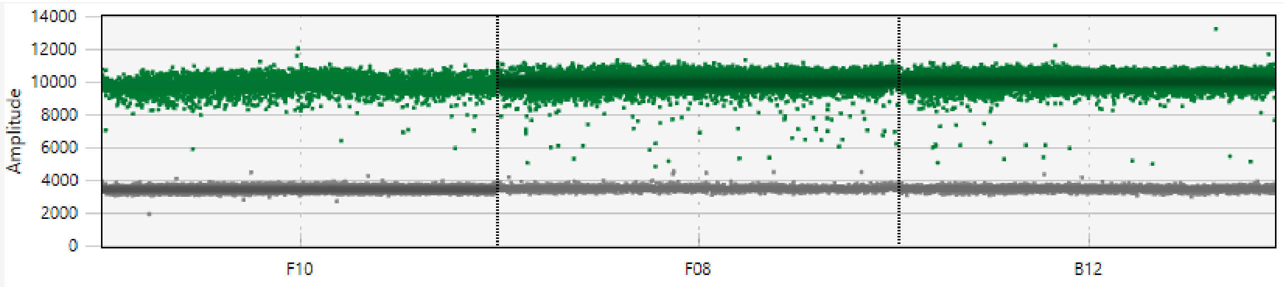

2D plots breakpoint 2 assay

Temporal pole

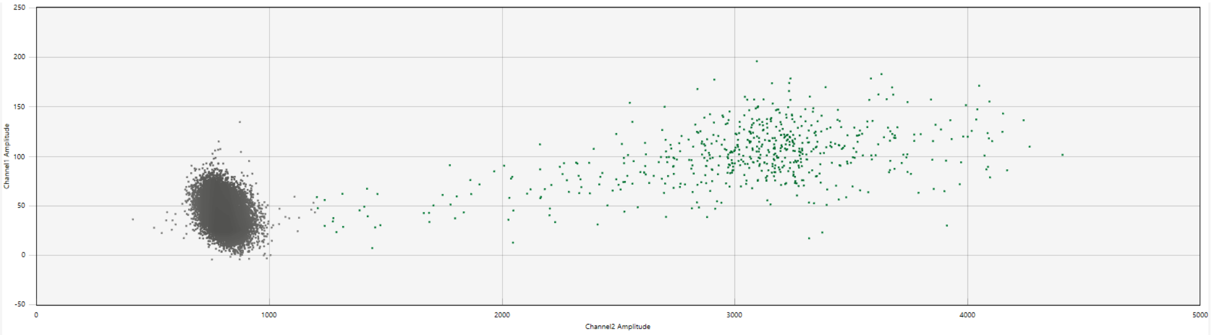

Liver right

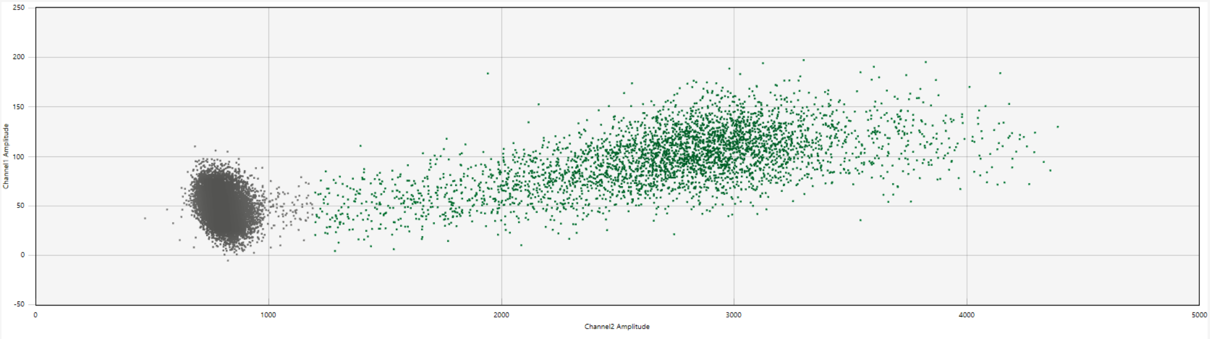

Left kidney

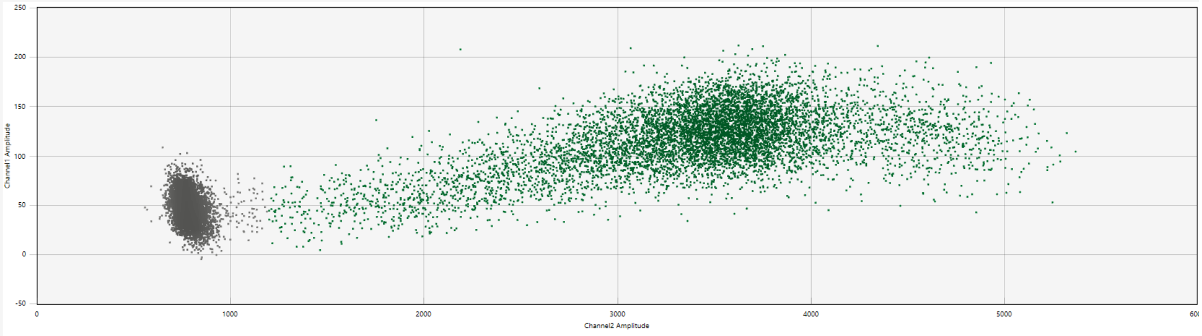

2D plots RPP30 assay (reference)

Temporal pole

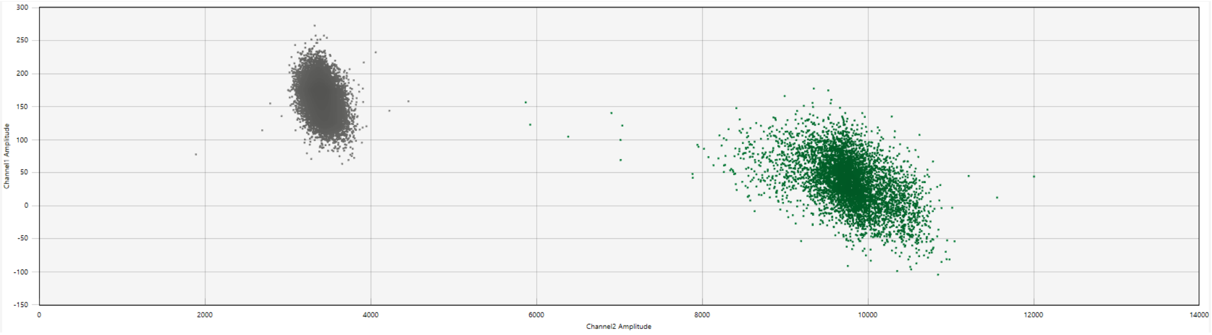

Liver right

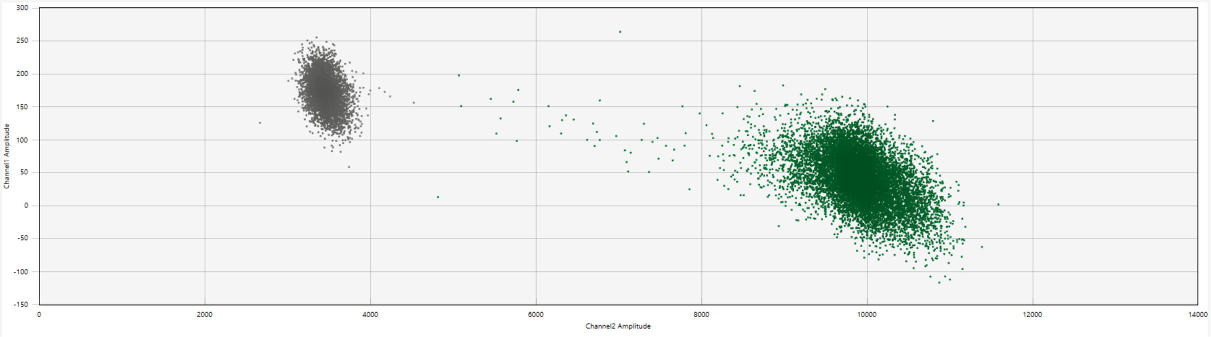

Left kidney

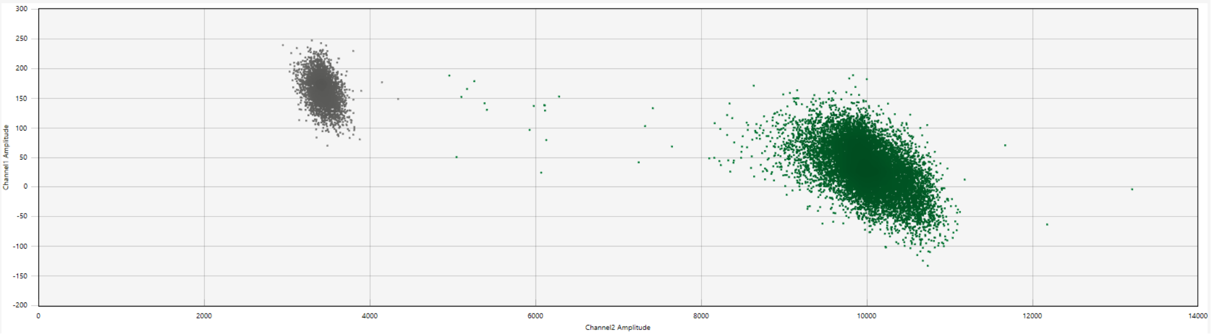

Thresholding

Automatic

Temporal pole

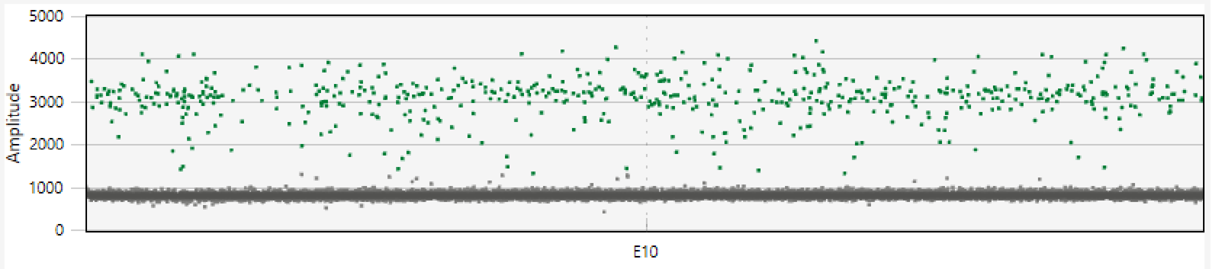

Conc copies: 44

Liver right

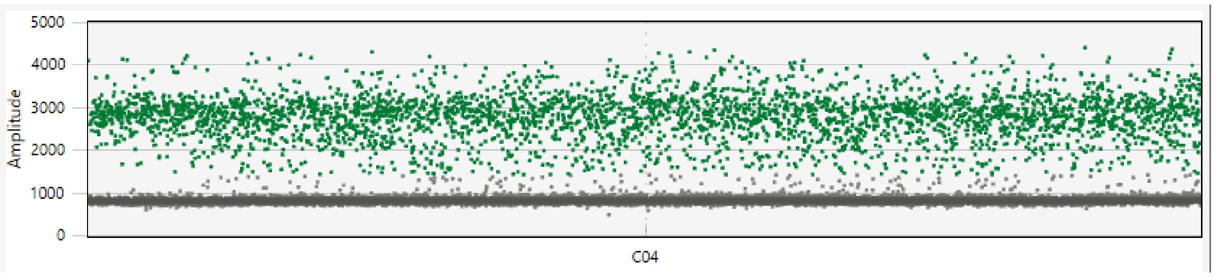

Conc copies: 291

Left kidney

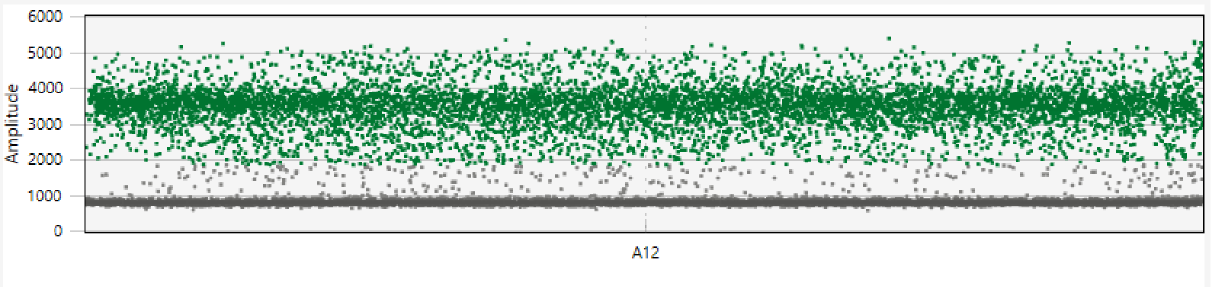

Conc copies: 854

Manual

Temporal pole

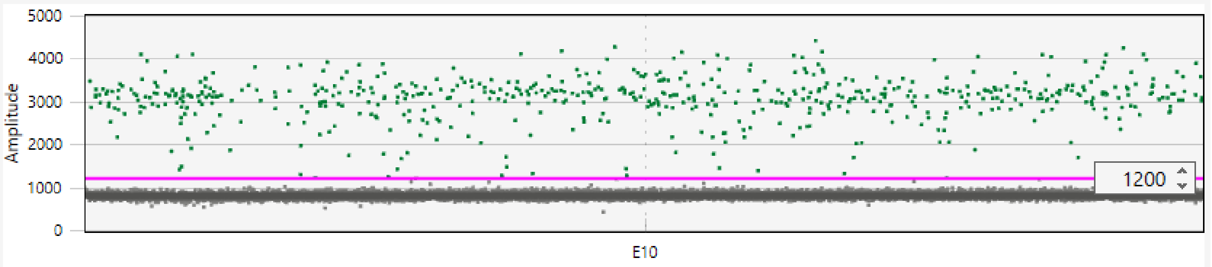

Conc copies: 44.6

Liver right

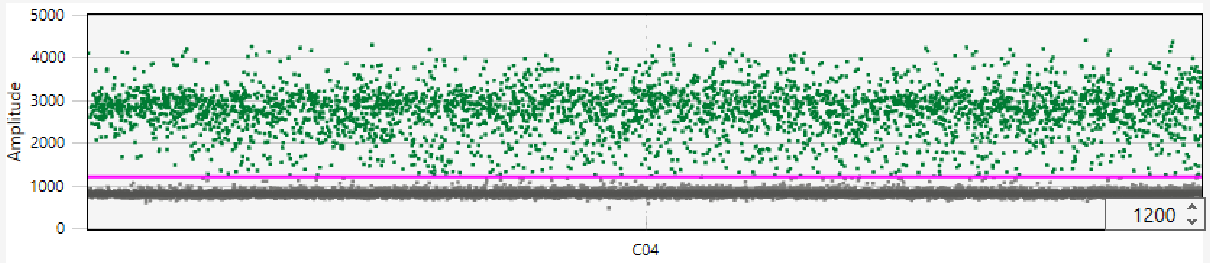

Conc copies: 298

Left kidney

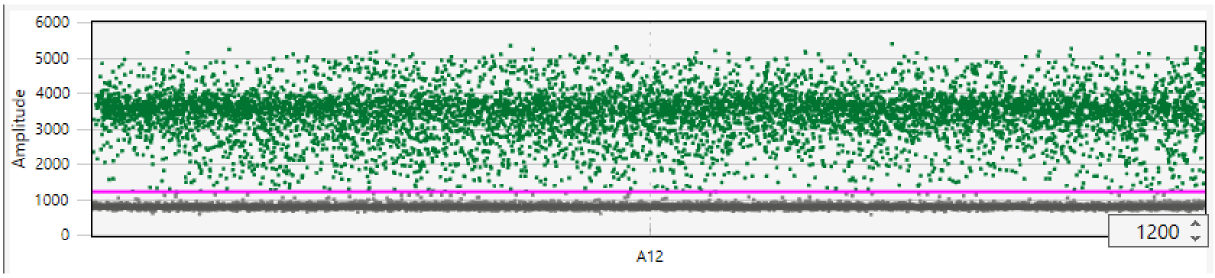

Conc copies: 898 copies/µL

Supplementary Table S2

Tissue	Tissue weight (mg)	DNA Qubit concentration ng/µl
Pancreas caput	67	99.8
Skeletal muscle (left thigh)	70	84.3
Thyroid (left)	53	42.6
Heart muscle left chamber	60	44.7
Heart muscle atrium	60	33.6
Heart muscle right chamber	50	20.0
Skin	29	4.84
Thalamus	65	7.42
Thyroid right	126	9.65
Fascia left thigh	20	6.75
Kidney right	87	80.2
Kidney left	100	81.7
Adrenal gland right white part	132	153
Adrenal gland left	144	80.4
Lymph nodule	93	61.6
Liver right	136	88.5
Pancreas corpus	87	23.5
Aorta	58	13.2
Vessel	36	22.4
Trachea white	10	43.5
Spleen	<10	221
Lung left	63.7	95.8
Lung right apical	127.8	50.5
Trachea red	80	19.7
Fat	10	9.32
Bone marrow	10	246
Adrenal gland right	81	82.4
Parietalcortex lateral	30	101
Frontal pole	30	28.6
Pariteal lobe medial	40	61
Substantia nigra	30	41.2
Cerebellum	20	123
Pituitary	10	104
Temporal pole	40	27.8
Amygdala	30	65.4
Olfactory bulb	10	40.5
Meningies	30	42.2
Gyrus singulus anterior	50	41.1
Insulate cortex	20	26
Occipital cortex	30	46.7
Nucleus lentiformis	30	13.2

Hippocampus	40	54.7
Nucleus caudatus	30	28.3

Supp Table 2: summary of tissue weight before DNA extraction and Qubit concentrations for all DNA. The mother underwent oophorectomy at the age of 46, and examination of paraffin-embedded material did not reveal any available egg cells for genetic analysis. All DNA from brain regions were collected from frozen, left side of the brain.
